# Normalization Methods on Single-Cell RNA-seq Data: An Empirical Survey

**DOI:** 10.3389/fgene.2020.00041

**Published:** 2020-02-07

**Authors:** Nicholas Lytal, Di Ran, Lingling An

**Affiliations:** ^1^ Interdisciplinary Program in Statistics, Statistical Bioinformatics Laboratory, University of Arizona, Tucson, AZ, United States; ^2^ Department of Epidemiology and Biostatistics, University of Arizona, Tucson, AZ, United States; ^3^ Statistical Bioinformatics Laboratory, Department of Biosystems Engineering, University of Arizona, Tucson, AZ, United States

**Keywords:** normalization, single-cell, comparison, RNA-seq, spike-in RNA

## Abstract

Data normalization is vital to single-cell sequencing, addressing limitations presented by low input material and various forms of bias or noise present in the sequencing process. Several such normalization methods exist, some of which rely on spike-in genes, molecules added in known quantities to serve as a basis for a normalization model. Depending on available information and the type of data, some methods may express certain advantages over others. We compare the effectiveness of seven available normalization methods designed specifically for single-cell sequencing using two real data sets containing spike-in genes and one simulation study. Additionally, we test those methods not dependent on spike-in genes using a real data set with three distinct cell-cycle states and a real data set under the 10X Genomics GemCode platform with multiple cell types represented. We demonstrate the differences in effectiveness for the featured methods using visualization and classification assessment and conclude which methods are preferable for normalizing a certain type of data for further downstream analysis, such as classification or differential analysis. The comparison in computational time for all methods is addressed as well.

## Introduction

Single-cell RNA sequencing (scRNA-seq) is a recent and powerful technology developed as an alternative to previously existing bulk RNA sequencing methods (Nawy, 2014). Bulk sequencing methods analyze the average genetic content for individual genes across a large population of input cells within a sample (e.g., a tissue), potentially obscuring transcriptional features and other differences among individual cells. Conversely, scRNA-seq is able to discern such heterogeneous properties within a sample (Patel et al., 2014) and has great potential to reveal novel subpopulations and cell types (Buettner et al., 2015). scRNA-seq has already been applied in the field of embryonic stem cell research, detecting developmental differences across weeks or days of growth (Macaulay and Voet, 2014). It has wide applications in cancer research as well, from identifying intratumor heterogeneity to profiling clonal evolution mechanisms (Zhang et al., 2016). However, individual cells have extremely tiny amounts of input material available, typically on the scale of picograms (Brennecke et al., 2013). The small scale of scRNA-seq input material means that some level of inaccuracy is inevitable even with the most precise instruments, resulting in an additional layer of stochasticity known as technical noise (Jia et al., 2017). During the sequencing process, reverse transcription is necessary to convert RNA to cDNA for use in amplification, but this introduces positional bias regardless of where the polymerization begins (Hebenstreit, 2012). The following amplification process counteracts low input material, though this in turn leads to additional bias as some genes may experience preferential amplification, leading to uneven representation in the data (Neu et al., 2017). The amplification process also runs the risk of producing dropout events, in which either genes known to be present in a sample are completely absent from the observed gene counts or genes are observed with lower value than their true expression (Van den Berge et al., 2018). These events frequently lead to excessive zeros, and in many cases, more than half of all counts. Traditional bulk approaches do not naturally accommodate these differences, and therefore lose their effectiveness when applied to scRNA-seq.

Normalization is critical to the development of analysis techniques on scRNA-seq and to counteract technical noise or bias. Before observed data can be used to identify differentially expressed genes or potential subpopulations, it must undergo these corrections, for what is observed is seldom exactly what is present within the data set. Over its years of development for scRNA-seq, several normalization methods have been utilized, ranging from variations of bulk sequencing methods to entirely new approaches designed specifically for single-cell studies. These methods frequently draw attention to the differences between technical noise (e.g., due to imprecise measurements) and biological/medical variation, attributed to natural differences among the cells under the same biological/medical condition (Kim et al., 2015). In this paper, the term “condition” refers to biological and medical conditions (e.g., cell type).

Although bulk-based normalization methods such as those included within differential expression analysis for sequence count data (DESeq) (Anders and Huber, 2010) and trimmed mean of M-values (TMM) (Robinson and Oshlack, 2010) have found varying degrees of success and early widespread use (Dillies et al., 2013), they do not effectively account for the limitations specific to scRNA-seq data. In particular, excessive zeros greatly impact DESeq due to its reliance on genes with nonzero counts in every cell, while TMM frequently overcorrects for scaling factor sizes (Vallejos et al., 2017). For these reasons, this paper focuses on normalization methods that have been specifically designed for scRNA-seq data, rather than adapted from bulk-based approaches.

Some scRNA-seq studies involve the use of spike-in molecules for the purpose of normalization (Jiang et al., 2011). The spike-in RNA set is artificially added to each cell's lysate in the same volume under the assumption that spike-ins and endogenous transcripts will experience similar variation among cells during the capture process (Lun et al., 2017). Since spike-in gene concentrations are known, normalization methods model existing technical variation by utilizing the difference between these known values and the values observed after processing. Spike-ins can also aid in quantifying capture efficiency to improve the quality of normalization (Stegle et al., 2015).

We compared seven existing normalization approaches that aim to reduce noise or bias specifically for scRNA-seq data: (1) Single-Cell Tagged Reverse Transcription (SAMstrt) (Katayama et al., 2013), (2) Bayesian Analysis of Single-Cell Sequencing Data (BASiCS) (Vallejos et al., 2015), (3) Gamma Regression Model (GRM) (Ding et al., 2015), (4) scran, a package for scRNA-seq data analysis (Lun et al., 2016), (5) Robust Normalization of Single-cell RNA-seq Data (SCnorm) (Bacher et al., 2017), and (6) Linnorm, a linear model and normality based normalizing transformation method for scRNA-seq data (Yip et al., 2017). To compare the effectiveness of these methods on data normalization, we considered two real data sets containing spike-in genes. Additionally, we compared normalization methods by using a simulation study, and lastly, we compared the three normalization approaches (Linnorm, SCnorm, scran) that do not require spike-in genes on a real data set consisting of human embryonic stem cells in various stages of the cell cycle, as well as on a 10X Genomics real data set of peripheral blood mononuclear cells (PBMCs).

## Materials and Methods

The focus of this study is comparing single-cell specific normalization methods. Of these, seven normalization techniques are defined and applied according to their respective packages. Information on the data sets used is listed in **Table 1**. Summary information for the normalization methods is listed in **Table 2**. As a note, variable names for some methods' formulas have been altered from their original forms for the sake of consistency in this review and the untouched forms can be found in the referenced papers. The corresponding R packages for all of these methods can be found in **Table 3**. All methods were performed using the default settings of their respective R packages.

**Table 1:** Summary of data sets used in this review.

Data Type	Author	Year	Protocol	Platform	# genes after cleaning	# cells after cleaning
Mouse embryonic stem cells	Islam et al.	2011	AbGene Thermo-Fast 96	Illumina Genome Analyzer IIx	11430	90
Mouse lung cells	Treutlein et al.	2014	Fluidigm C1	Illumina HiSeq 2000	12073	186
Human embryonic data	Leng et al.	2015	Fluidigm C1	Illumina HiSeq 2500	19084	247
PBMC data	Zheng et al.	2017	10X Genomics	GemCode	13714	2649

**Table 2:** Summary of normalization methods, including the basic description and whether the method uses spike-in genes information.

Method	Author	Year	Spike-ins	Model Description
SAMstrt	Katayama et al.	2013	Yes	Poisson resampling and non-parametric statistics
BASiCS	Vallejos et al.	2015	Yes	Use spike-ins for hierarchical Poisson/Gamma model for technical variability. Expand model to incorporate biological genes with new Poisson model
GRM	Ding et al.	2015	Yes	Gamma regression model from spike-ins
Simple Norm.	Satija et al.	2015	No	Divide gene counts for cells, then multiply by scale factor and apply a log(x+1) transformation to the result (included in the Seurat package as NormalizeData)
scran	Lun et al.	2016	No	Deconvolution of size factors from constructed linear system. Form pools of cells and normalize using summed expression values
SCnorm	Bacher et al.	2017	Optional	Quantile based model for log sequencing depth.
Linnorm	Yip et al.	2017	Optional	Linear models defined with a normalization strength coefficient to update means. Focuses on stable genes to perform normalization

**Table 3:** A list of source packages for downloading.

Package	Source
SAMstrt v.0.99.0	(https://github.com/shka/R-SAMstrt/archive/0.99.0.tar.gz)
BASiCS v.1.0.1	Bioconductor (http://www.bioconductor.org)
GRM v.0.2.1	(http://wanglab.ucsd.edu/star/GRM/)
scran v.1.6.9	Bioconductor (http://www.bioconductor.org)
SCnorm v.1.0.0	Bioconductor (http://www.bioconductor.org)
Linnorm v.2.2.0	Bioconductor (http://www.bioconductor.org)
Seurat v.3.0.0	CRAN (https://CRAN.R-project.org/package=Seurat)

### Single-Cell Tagged Reverse Transcription (SAMstrt)

SAMstrt is an adaptation of SAMseq designed for the single-cell tagged reverse transcription (STRT) sequencing method (Islam et al., 2012). SAMseq is a nonparametric approach to differential expression in bulk sequencing, designed to be resistant to outliers (Li and Tibshirani, 2011). It uses the two-sample Wilcoxon test and Poisson resampling to detect the genes associated with an outcome in RNA-Seq or other sequencing-based comparative genomic experiments. SAMstrt is developed on this method and alters the sequencing depth estimation by assuming equivalent spike-in-molecules per cell in each condition. In other words, SAMstrt builds on the method by accounting for and correcting differences in sequenced spike-in reads across cells, even in the presence of highly variable sequencing depths. SAMstrt is effective at identifying highly expressed features, though it depends on the number of total reads per spike-in gene across all cells, with higher performance expected for deeper sequencing depth.

### Bayesian Analysis of Single-Cell Sequencing Data (BASiCS)

BASiCS uses a fully Bayesian approach that separates data variation into gene-specific constants, technical noise, and biological variation. Though using spike-in genes, it does not rely on them exclusively to determine technical variation, instead electing to jointly model spike-in genes and biological genes (i.e., all other genes in the data set).

For normalization, BASiCS first utilizes the spike-in genes to determine a hierarchical model for technical variation, with *X_ij_* representing the expression count of spike-in gene *i* in cell *j*, using the Poisson and Gamma distributions. Here, for cell *j μ_i_* is the normalized expression rate of gene *i* from the same cell type, *v_j_* is a random effect centered on the capture efficiency constant *s_j_* and *θ* quantifies technical noise using information from all genes and all cells. Once established for the spike-in genes, this model expands to account for the biological genes:

(1) $$X_{ij}|\mu_{i},\phi_{j},\nu_{j},\rho_{ij}∼\{\begin{array}{c} Poisson\left(\phi_{j}\nu_{j}\mu_{i}\rho_{ij}\right),\;\;for\;biological\;genes\\ Poisson\left(\nu_{j}\mu_{i}\right),\;\;for\;spike-in\;genes \end{array}$$

(2) $$with\;v_{j}|s_{j},\theta ∼Gamma\;\left(\frac{1}{\theta },\frac{1}{\left(s_{j}\theta \right)}\right)\;and\;\rho_{ij}|\delta_{i}∼Gamma\;\left(\frac{1}{\delta_{i}},\frac{1}{\delta_{i}}\right)$$

In this equation, *ρ_ij_* represents a mutually independent set of random effects from *v_j_*, while including cell-specific size factors *ϕ_j_*. Note that cell-size factors are only applicable to the biological genes in this equation; spike-in genes are included in equal amounts for each cell, regardless of cell-size, so spike-in genes are independent of such effects. BASiCS treats cell-specific normalizing constants (*ϕ_j_* and *s_j_*) as model parameters and estimates them by combining information across all genes. Properly cleaning data by removing genes and cells with all zero or very low total read counts (<10 for genes, <10000 for cells) is especially important for BASiCS.

### Gamma Regression Model (GRM)

The Gamma Regression Model uses spike-in genes to construct a model that accounts for differences between observed and known spike-in concentrations. Notably, it predicts concentration as a function of the fragments per kilobase of transcript per million mapped reads (FPKM).

To account for a wide range of gene concentrations, GRM employs the gamma regression model to fit the log transformed concentration data against the log transformed FPKM reads for the spike-in genes. Due to the non-linearity of the reads, they are modeled using a polynomial function *μ*(*X*) that can be adjusted to various degrees (*D*_0_ = 1~ 4) to find an optimal fit with minimal average technical noise of spike-in genes. Furthermore, GRM is applied to cells individually rather than simultaneously, unlike other normalization methods. Once the regression model is formed for an individual cell, it calculates the expectation of the biological gene concentration based on the given FPKM as follows:

(3) C∼Gamma(μ(X),ϕ)

(4) $$\mu (X)=\sum_{d=0}^{D_{0}}\beta_{d}X^{d}$$

(5) $$f\left(C\right)=\;\frac{1}{C\text{Γ}\left(\phi \right)}{\left(\frac{\phi C}{\mu \left(X\right)}\right)}^{\phi }exp\left(-\frac{\phi C}{\mu \left(X\right)}\right)$$

where *X* = log(FPKM), *C* = log(concentration). The parameters are determined using maximum likelihood estimation for Gamma regression. The normalized expression for gene *i* can be calculated for the observed gene expression by:

(6) $$Y_{i}=E(C_{i})=\hat{\mu }(X_{i})=\sum_{d=0}^{D_{0}}{\hat{\beta }}_{d}X_{i}^{d}$$

GRM is a method that performs normalization and denoising for individual cells. As such, it is primarily used for denoising effect, as it does not perform normalization directly among cells.

### scran—Methods for Single-Cell RNA-Seq Data Analysis

The scran method uses a deconvolution approach that partitions cells into pools, normalizes across cells in each pool, then uses the resulting system of linear equations to define individual cell factors. This method takes extra steps to counteract assumptions about differential expression (DE) genes by estimating a size factor term for each cell pool and utilizing these estimates to approximate a size factor term for individual cells. Cell pools are designed to have comparable library sizes to account for potential estimation errors. By keeping sizes consistent, estimation errors are less likely to disproportionately affect extreme cells under the same condition.

For *X_ij_*, the count of a non-DE gene *i* in cell *j* the expected value is *E*(*X_ij_*)=*θ_j_λ_i_*_0_, where *θ_j_* is the cell-specific bias, and *λ_i_*_0_ is the expected gene expression count. Including a constant adjustment factor *t_j_* for cell *j*, the normalized expression value *Y_ij_* is defined as $Y_{ij}=X_{ij}t_{j}^{-1}$ with expectation of $E\left(Y_{ij}\right)=\theta_{j}\lambda_{i0}t_{j}^{-1}$. Now for pool *k* of arbitrary cell set *S_k_* let *V_ik_* be the sum of *Y_ij_* across all cells in the set, *U_i_* be the mean of *Y_ij_* across the entire data set of N cells, and *R_i__k_* be the ratio of *V_i__k_* to *U_i_*, so:

(7) $$E(V_{ik})=\lambda_{i0}\sum_{j\in S_{k}}\theta_{j}t_{j}^{-1}$$

(8) $$E(U_{i})=\lambda_{i0}N^{-1}\sum_{j\in S_{0}}\theta_{j}t_{j}^{-1}$$

(9) $$E\left(R_{ik}\right)\approx \frac{E\left(V_{ik}\right)}{E\left(U_{i}\right)}=\frac{\sum_{S_{k}}\theta_{j}t_{j}^{-1}}{N^{-1}\sum_{S_{0}}\theta_{j}t_{j}^{-1}}=\frac{\sum_{S_{k}}\theta_{j}t_{j}^{-1}}{C}$$

with set *S*_0_ of all cells in the entire data set and constant *C* independent of the gene, cell, and pool. This results in the pool-based size factor *R_i__k_*, whose estimates can be applied to individual cells accurately. Linear equations are then set up for every pool using the estimate of the size factor and treating $\theta_{j}t_{j}^{-1}$ is unknown, using least-squares methods to solve for all cells. The pool sizes chosen for scran are restricted by the number of cells in each condition within a data set. The authors recommended that the number of cells is at least 20, though smaller sizes may be possible. A larger number of cells will also lead to computational complexity, so this may impact the execution time for certain data sets.

### Robust Normalization of Single-Cell RNA-Seq Data (SCnorm)

This recently developed method does not rely on global scaling factors that many other methods use for normalization. Instead, it focuses on two layers of quantile regression to effectively group genes and estimate their dependence. While it does not require spike-ins, SCnorm can use them to improve its accuracy, provided the spike-in genes cover a similar range of gene expression to that of the biological genes in the study.

For SCnorm, let *X_i,j_* be the log nonzero expression count for gene *i* in cell *j*, and *D_j_* as the log sequencing depth for cell *j*. Furthermore, the total number of pools *K* for which the count-depth relationship substantially varies is chosen, starting at *K*=1. The gene-specific relationship between log of raw expression and log sequencing depth with median quantile regression is as follows:

(10) $$Q^{0.5}\left(X_{i,j}|D_{j}\right)=\beta_{i,0}+\beta_{i,1}D_{j}$$

However, the median may not be an appropriate representation depending on the gene, so this can be extended to multiple quantiles *τ* and polynomial degrees *d* for all the genes in the current pool *k,* where *k*={1,..,*K*}:

(11) $$Q^{\tau_{k},d_{k}}\left(X_{j}|D_{j}\right)=\beta_{0}^{\tau_{k}}+\beta_{1}^{\tau_{k}}D_{j}+\cdots +\beta_{d}^{\tau_{k}}D_{j}^{d_{k}}$$

SCnorm also designates ${\hat{\eta }}_{1}^{\tau_{k}}$ as the estimated count-depth relationship of expression values from the median first-degree polynomial:

(12) $$Q^{0.5}\left({\hat{X}}_{j}^{\tau_{k}}|D_{j}\right)=\eta_{0}^{\tau_{k}}+\eta_{1}^{\tau_{k}}D_{j}$$

Let $\tau_{k}^{*}$ and $d_{k}^{*}$ be the specific values that minimize the absolute difference between the estimate count-depth relationship and the mode of the estimate of slopes in (Equation 10) across all genes. The scale factor *SF_j_* for cell *j* is defined as below, as are the normalized counts *Y_i,j_*:

(13) $$SF_{j}=\frac{e^{{\hat{X}}_{j}^{\tau_{k}^{*},d_{k}^{*}}}}{e^{X^{\tau_{k}^{*}}}}$$

(14) $$Y_{i,j}=\frac{e^{X_{i,j}}}{SF_{j}}$$

SCnorm begins with *K*=1 and calculates the modes of slopes for median quantile regression within a certain number (default = 10) of pools of equal size. If any mode exceeds 0.1, the normalization is deemed insufficient, and *K* increases by 1 to repeat the process.

SCnorm sequentially chooses the number (*K)* of pools for which the count-depth relationship varies significantly. Depending on the nature of the data set, the number of pools necessary can greatly vary, and data sets of similar size may have noticeably different execution times as a result. In particular, exceptionally large amounts of zeros in the data may require a large number of pools to converge, increasing total computational time.

### Linear Model and Normality Based Normalizing Transformation Method (Linnorm)

Linnorm is a newer method designed for both normalization and transformation of scRNA-seq data, though for the purpose of this paper we consider the normalization portion only. It first identifies stable genes (i.e., exhibiting nearly zero variance across all cells, according to the referenced paper) with an initial normalization step that converts initial expression level *X_ij_* for gene *i* and cell *j* to relative scale *R_ij_* as follows:

(15) $$R_{ij}=\frac{X_{ij}}{\sum_{i=1}^{m}X_{ij}}\left(1\leq i\leq m,\;1\leq j\leq n\right)$$

When only performing normalization, Linnorm then defines the expression level *G_ij_* as

(16) $$G_{ij}=ln\left(\lambda R_{ij}\right)$$

where *λ* is the median of total counts across all cells and employs the expression means *z_i_* within *n* linear models of the form:

(17) $$z_{i}=a_{j}X_{ij}+b_{j}$$

in which the parameters *a* and *b* in each model are estimated through the linear model. Then, the normalized data can be obtained using the normalization strength coefficient *c* (0≤*c*≤1), set to 0.5 by default in the following process:

(18) $$\;a_{j}^{updated}=c\left(a_{j}-1\right)+1$$

(19) $$b_{j}^{updated}=b_{j}*c$$

(20) $$B_{ij}=exp\left(a_{j}^{updated}G_{ij}+b_{j}^{updated}\right)$$

(21) $$Y_{ij}=ln\left(B_{ij}+1\right)$$

Linnorm depends on the assumption that genes are homogeneously expressed across different cells, and genes with low expression can introduce skewness to data that may violate these assumptions. Also, though it relies on only stable genes to determine normalization parameters, these parameters are applied universally, which may introduce additional variation for less stable genes.

### Simple Normalization

In addition to the above methods, we obtain a baseline comparison for normalization through the use of the Seurat (Satija et al., 2015) R package's NormalizeData function. This method, referred to as “Simple Norm” in subsequent plots, is a global normalization process that by default divides gene counts for a cell before multiplying by the scale factor and natural log transforming the result with log(x+1) to account for zero counts.

### Bulk Normalization Methods

Although this paper is focused on the performance of single-cell-based normalization methods, an additional section comparing the performance of two popular bulk-based normalization methods is included. The “median of ratios” (MoR) approach is analyzed using version 1.17.15 of the DESeq2 package, while the “trimmed mean of M values” (TMM) approach is analyzed using version 3.19.3 of the edgeR package. Analysis for bulk methods can be found in section 4 of the **Supplementary Materials**.

### Data

For spike-in gene case, we chose two scRNA-seq mouse data sets, one on embryonic stem cells and fibroblasts and the other on four development stages of epithelial lung cells. We also simulated a data set based on the stem cell study. For non-spike-in gene case, we utilized a human data set with three cell-cycle states.

#### 1) Mouse Embryonic Data

The first data set, drawn from a study by Islam et al. and hereafter referred to as the mouse embryonic data, has two groups of cells: 48 mouse embryonic stem cells (ES) and 44 mouse embryonic fibroblasts (MEF) (Islam et al., 2011). Counts for this data can be found at the GEO Database under accession number GSE29087. This data set also includes a set of 8 spike-in genes (synthetic control mRNAs).

#### 2) Mouse Lung Data

The second spike-in real data set originates in a study by Treutlein et al. (2014), hereafter referred to as the mouse lung data, and consists of mouse lung epithelial cell data separated into three stages of embryo development labeled by number of days: 45 E14.5 cells, 27 E16.5 cells, and 80 E18.5 cells, plus 46 adult cells. Counts for this data can be found at the GEO Database under accession number GSE52583. Additionally, this data includes a set of 92 ERCC spike-in genes, available through Thermo Fisher Scientific's Ambion line of spike-in control mixes (ThermoFisher Scientific, 2015) (https://tools/thermofisher/com/content/sfs/manuals/ERCC92.zip).

#### 3) Simulated Data

The simulated data set is based on the mouse embryonic data. Using a similar approach to anexisting study on scRNA-seq simulation for normalization (Vallejos et al., 2017), we divided the first group of mouse embryonic stem cells into two subgroups of equal size that are then differentiated by upregulating random genes between the two subgroups. Based on the mean counts for genes across all cells, global scaling factors for each gene, and the overdispersion of each gene, we then generated new read counts through a negative binomial distribution, mimicking scRNA-seq data. In this instance, global scaling factors refer to constant factors by which expression within each cell is scaled in order to remove cell-specific biases (Vallejos et al., 2017). These factors assume read counts in a cell have an expected value proportional to both gene-specific expression and cell-specific scaling factors. We also subject one of the two subgroups to an n-fold change [n=(2, 5)] in expression among a random selection of 1000 genes. This allows us to observe each method's sensitivity to various levels of differential expression.

#### 4) Human Embryonic Data

In addition to the data sets with provided spike-in genes, we selected a data set specifically for the methods that do not use spike-in genes to provide a closer comparison. This data set belongs to the H1-FUCCI case study by Leng et al. (2015), hereafter referred to as the human embryonic data, and contains 247 human embryonic stem cells (hESC) separated by cell-cycle state: 76 G2 cells, 80 S cells, and 91 G1 cells. Counts are available at the GEO Database under accession number GSE64016.

#### 5) Peripheral Blood Mononuclear Cell (PBMC) Data

To demonstrate the effectiveness of these methods with more recent protocols, we selected thisdata set, hereafter referred to as the PBMC data. This data set is a representative of 10X Genomics's more recent GemCode platform, which several scRNA-seq studies have adopted, and which typically processes much larger numbers of cells at more sparse count levels. As with the human embryonic data set, there are no spike-in genes present, and we again compare only methods that do not use spike-in genes. The data set consists of 2700 PBMCs (2649 of which were used in this study) and is part of a larger dataset used in a study by Zheng et al. (2017), with the original data set consisting of 68k PBMCs from a healthy donor. Cell groups for this dataset were determined through a clustering pipeline as part of the Seurat R package. Counts are available at the NCBI Sequence Read Archive under accession number SRP073767, and the smaller version used in this study can be found at http://support.10xgenomics.com/single-cell/datasets.

## Results

The chosen normalization methods were first compared on two mouse scRNA-seq data sets. For further comparisons, we also constructed a simulated data set based on one of the real sets. We also include a human data set with three cell-cycle states to test methods not dependent on spike-in genes. The raw data are noisy and must be cleaned before subjecting the data to normalization. Details for the basic cleaning process can be found in the **Supplementary Materials**. All final plots after normalization are at a log-transformed scale, as employed in similar studies (Chapman et al., 2002; Lin et al., 2017).

### Visualization Analysis

Principal component analysis, a linear method of dimensionality reduction, is used to demonstrate differences among cells under different conditions within each data set (Wold et al., 1987). By projecting the data through orthogonal transformations, PCA allows most of the variance in a data set to be contained within the first few principal components. Very often, by plotting the first two principal components, we can observe separation within the data from a PCA visual representation of the scRNA-seq data. However, since PCA is a linear method in dimension reduction, it is not always able to capture differences among cell groups. As such, we consider t-SNE analysis, a non-linear method for visualization (Van Der Maaten and Hinton, 2008). This non-parametric method for dimension reduction is a modern alternative to PCA that displays patterns for visualization in just a few dimensions—all t-SNE analysis is performed within the Rtsne() package, version 0.13. Additionally, we employ UMAP (Becht et al., 2019) analysis, a new non-linear alternative to t-SNE that is resistant to loss of large-scale information and suitable for very large datasets. All UMAP analysis is performed with the runUMAP() function within the Seurat package, version 3.0.0. The following visualization plots focus on the t-SNE results, while PCA and UMAP results can be found in the **Supplementary Materials**.

### Visualization of Data Set 1): Mouse Embryonic Data

**Figure 1:**
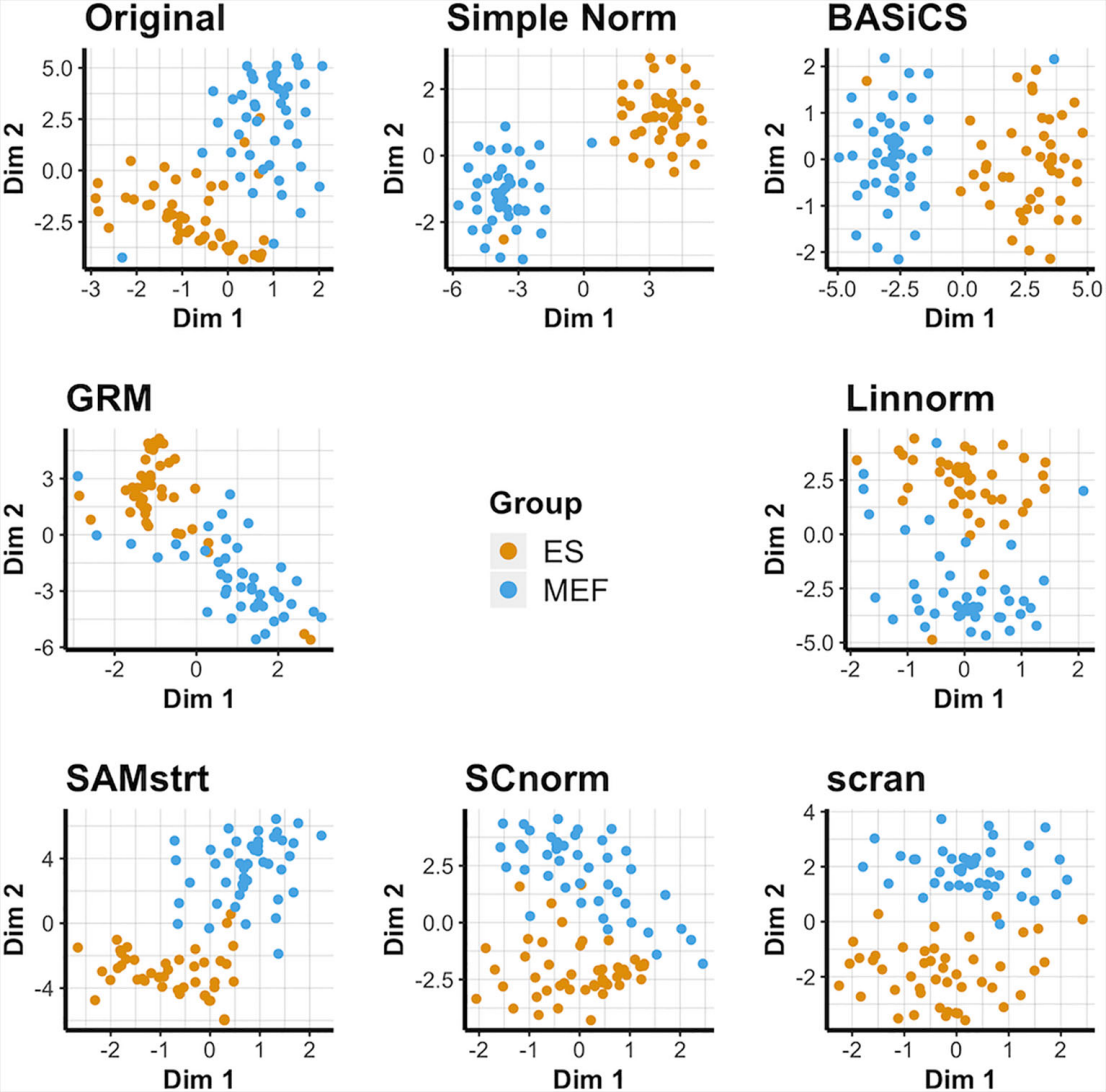
t-SNE plots of the mouse embryonic data set under various normalization methods, with the original data for comparison.

The results for the t-SNE visualization are displayed in **Figure 1**. Of the normalization methods, BASiCS and simple normalization present the clearest division between the two cell types. In comparison, SAMstrt, scran, and SCnorm form groups with mild overlap. Linnorm and GRM have several misclassified points despite forming two main groups.

PCA plots suggest the different cells do naturally form separate groups, but there is relatively little separation for some cells compared with others (**Supplementary Figure 1**). Most of the normalization methods succeed to varying degrees in forming separate groups, though occasional outliers lead to some cells being grouped incorrectly. However, the limitations of PCA are especially clear for the GRM method, which forms an exponential model from spike-ins and is not effectively represented by PCA's linear approach due to the presence of some abnormally large outliers. UMAP plots (**Supplementary Figure 2**) present an alternative conclusion in which SAMstrt and simple normalization form the most distinct groups, though still with some misclassified cells.

### Visualization of Data Set 2): Mouse Lung Data

**Figure 2:**
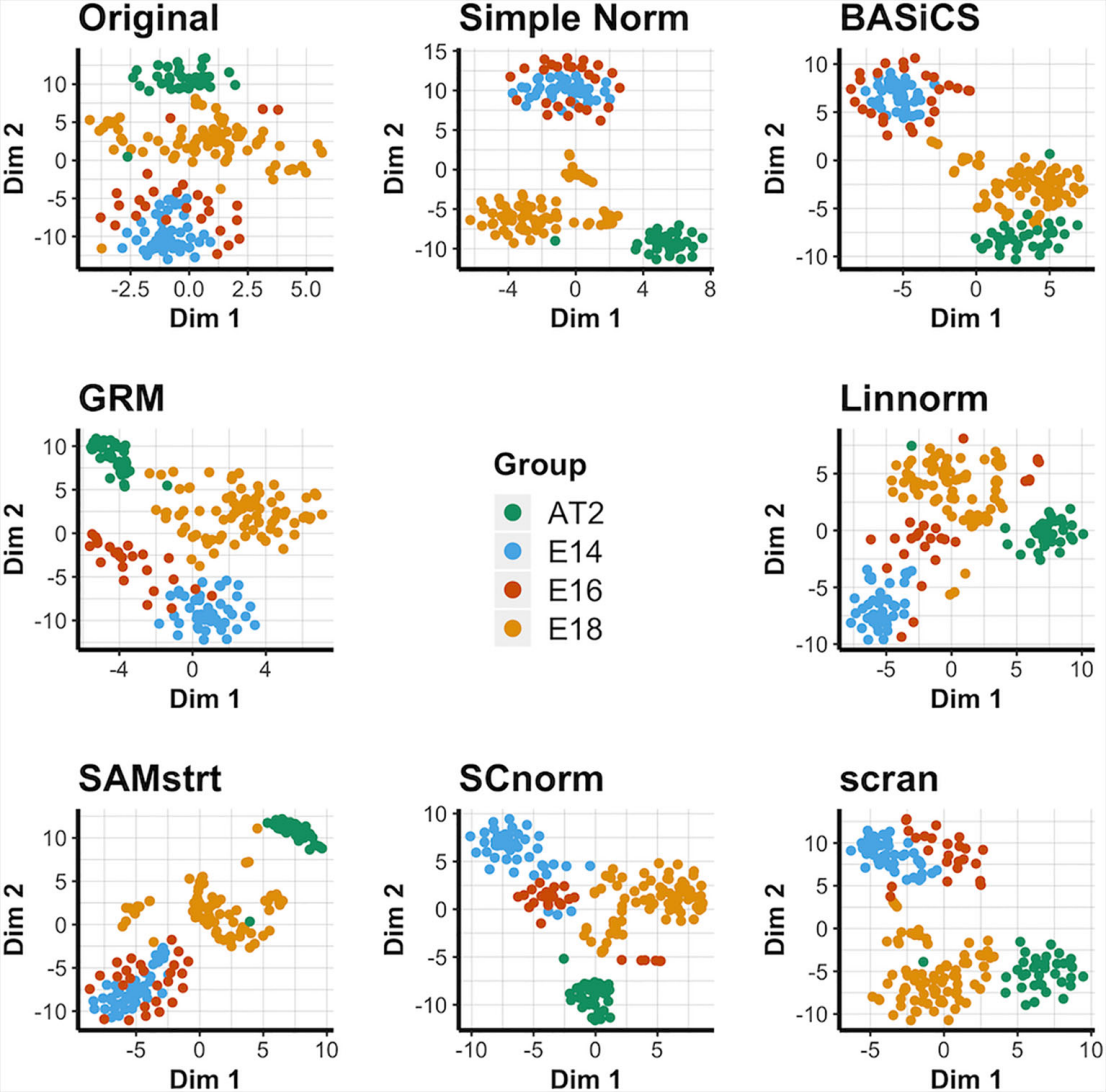
t-SNE plots for the mouse lung data set under various normalization methods, with the original data for comparison.

For the four conditions in the mouse lung data set, the original data contains considerable overlap among groups, and the normalization methods each maintain this overlap in some form (**Figure 2**). It is especially common to merge the E14 and E16 conditions together, suggesting a lack of strong differentiation between the two conditions during this range of embryonic development. BASiCS, SAMstrt, scran, and simple normalization are unable to separate the E14 and E16 conditions. Here, Linnorm, GRM, and SCnorm are more effective at separating the two conditions, though there is still a large amount of overlap for a number of cells. The PCA plots yield similar results (**Supplementary Figure 3**), with no methods able to form truly distinct groups for conditions. UMAP plots (**Supplementary Figure 4**) further demonstrate the difficulty of separating the two conditions, with no method truly being able to distinguish E14 from E16 cells.

### Visualization of Data Set 3): Simulation Data

**Figure 3:**
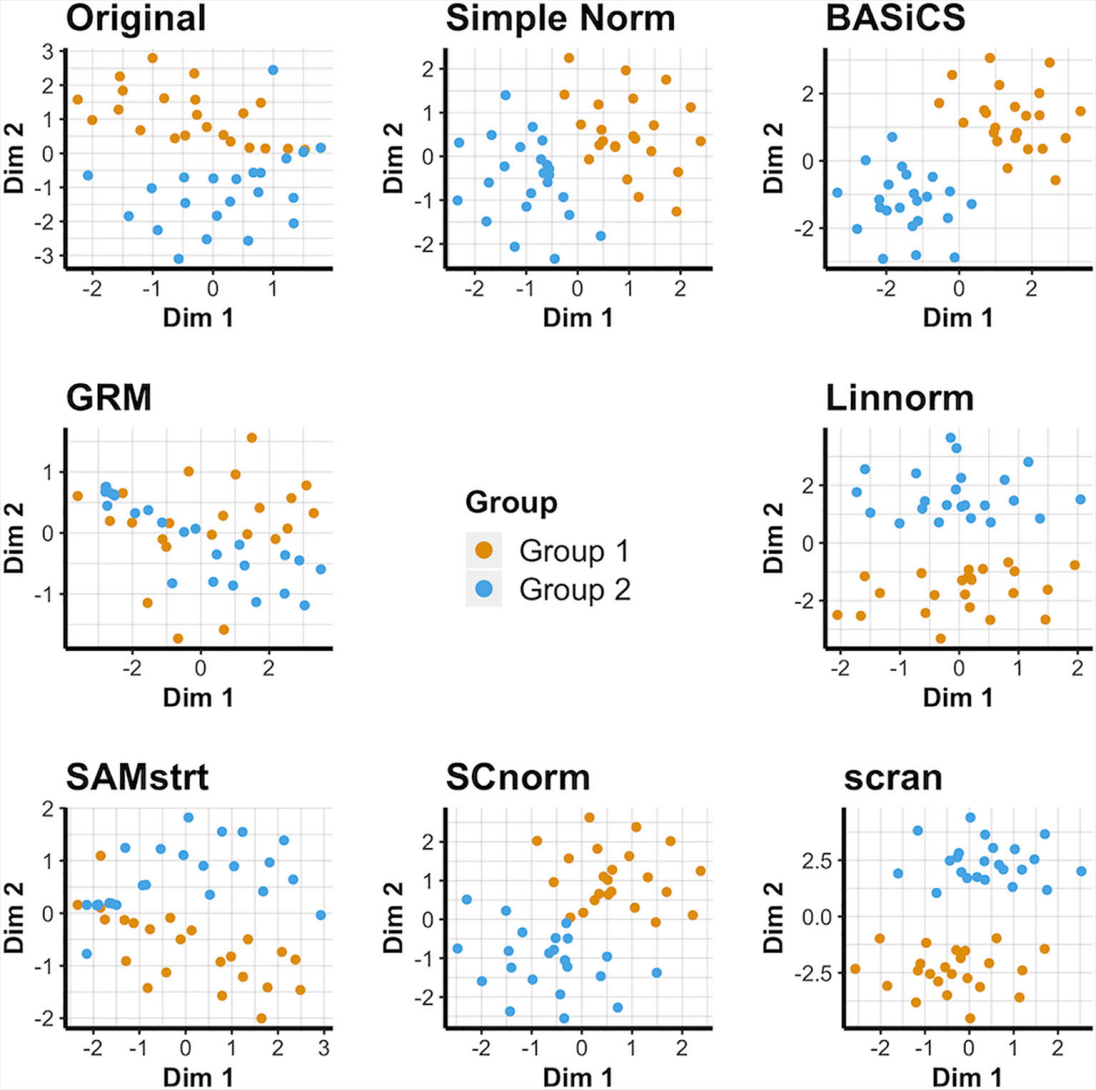
t-SNE plots for the 2-fold change simulated data set under various normalization methods, with the original data for comparison.

Simple normalization and the scran and BASiCS methods all form distinct groups for both the 2-fold change data set (**Figure 3**) and the 5-fold change data set (**Supplementary Figure 5**), even when relatively few distinctions remain between the two cell groups. Conversely, GRM and SAMstrt both struggle at separating these two groups for the simulated data sets. SCnorm and Linnorm both somewhat separate the groups for both 2-fold and 5-fold change data.

For the PCA plots, it is obvious that most normalization methods result in a clear separation between the cell groups for 2-fold change data (**Supplementary Figure 6**), with the exception of GRM and SAMstrt. Conclusions are consistent for the 5-fold change data (**Supplementary Figure 7**). This is attributed to the linear relationship resulting from gene upregulation between two subgroups when constructing the simulated data. UMAP plots (**Supplementary Figures 8**, **9**) confirm this and demonstrate the difficulties GRM and SAMstrt have in cleanly identifying cells between the two groups in comparison.

### Visualization of Data Set 4): Human Embryonic Data

**Figure 4:**
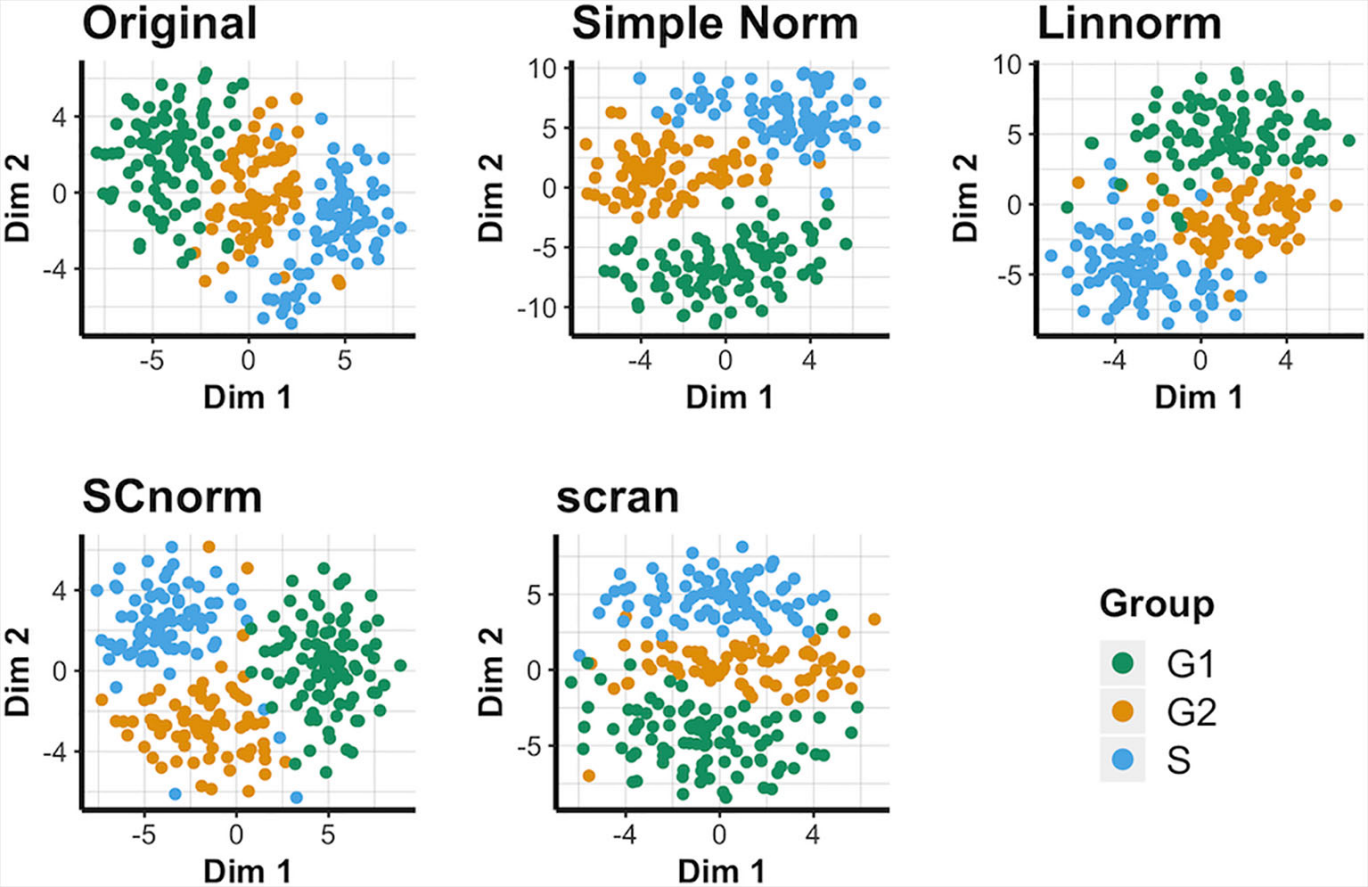
t-SNE plots for the human embryonic data under various normalization methods.

This data set does not contain spike-in genes, so only methods not reliant on spike-in genes are compared for this data (i.e. Linnorm, SCnorm, and scran). The t-SNE results (**Figure 4**) suggest that when spike-ins are not available, SCnorm is the most effective at separating the three cell-cycle groups into generally distinct groups. However, Linnorm and scran are less effective at separating the groups. PCA plots fail to reveal any outstanding performers for this data set (**Supplementary Figure 10**). No methods can separate conditions very well, though SCnorm comes closest to doing so. UMAP plots (**Supplementary Figure 11**) are similar, though notably simple normalization appears most effective at maintaining all S cells as a single cluster, rather than splitting them into two as other methods do.

### Visualization of Data Set 5): PBMC Data

**Figure 5:**
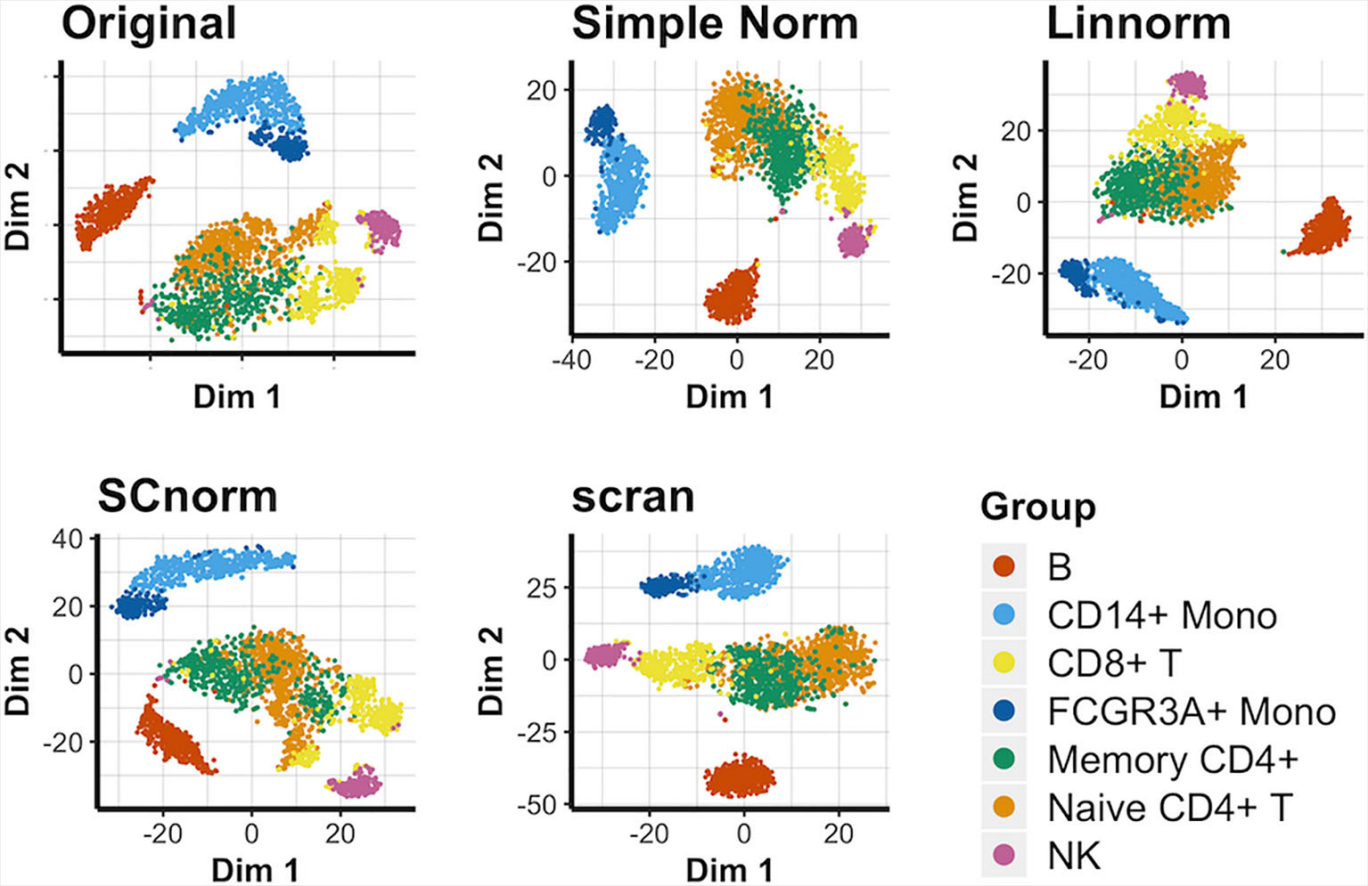
t-SNE plots for the PBMC data under various normalization methods.

We again compare methods not reliant on spike-in genes for this data set (**Figure 5**). In this case, most approaches, including simple normalization, appear to be comparable in effectiveness, with no approach able to completely separate cell groups in comparison to the original data. Existing similarities among the cell types depicted highlight the important of individual marker genes in distinguishing groups from each other. These observations hold true for the PCA plots (**Supplementary Figure 12**) and UMAP plots (**Supplementary Figure 13**) as well. In addition, we consider a smaller subset of this data set consisting of solely the T-cell categories within the PBMC data set to better observe how well each normalization method can separate similar cell types. The resulting t-SNE plot (**Supplementary Figure 14**) suggests that none of the viable non-spike-in methods are able to definitively succeed as separating the similar T-cell categories.

For all data sets, we also attempted sparse PCA (Zou et al., 2006), a variant that relies on fewer input variables to produce its principal components. This accounts for a large number of zero counts presenting in the data, allowing construction of principal components even in the absence of certain genes. However, we found that the first two principal components did not differ significantly from regular PCA and did not include the resulting plots.

### Classification Analysis

Although visualization methods give a direct impression of cell relationships, quantitative metrics are necessary to evaluate the performance of these normalization methods. We performed classification analysis to assess the effectiveness of the methods. There are various classification methods, e.g., linear discriminant analysis (LDA) (Fisher, 1936), support vector machines (SVM) (Cortes and Vapnik, 1995), and K-nearest neighbors (KNN) (Altman, 1992). We used KNN for evaluating the performance of normalization methods due to its non-parametric nature and ability to naturally work beyond two-group classification. We then used Cohen's statistic (Cohen, 1960) instead of misclassification rate as a measure of effectiveness. By adjusting the diagonal in a confusion matrix for agreement by chance, Cohen's kappa more accurately describes agreement in the case of unequal group sizes, which is common for scRNA-seq studies. The details of classification analysis can be found in the **Supplementary Materials**. We also considered the use of Random Forest classifiers (Breiman, 2001) based on those included as part of the Seurat package, though we found the results were generally comparable and did not reveal any new or conflicting findings compared to our initial approach.

**Figure 6:**
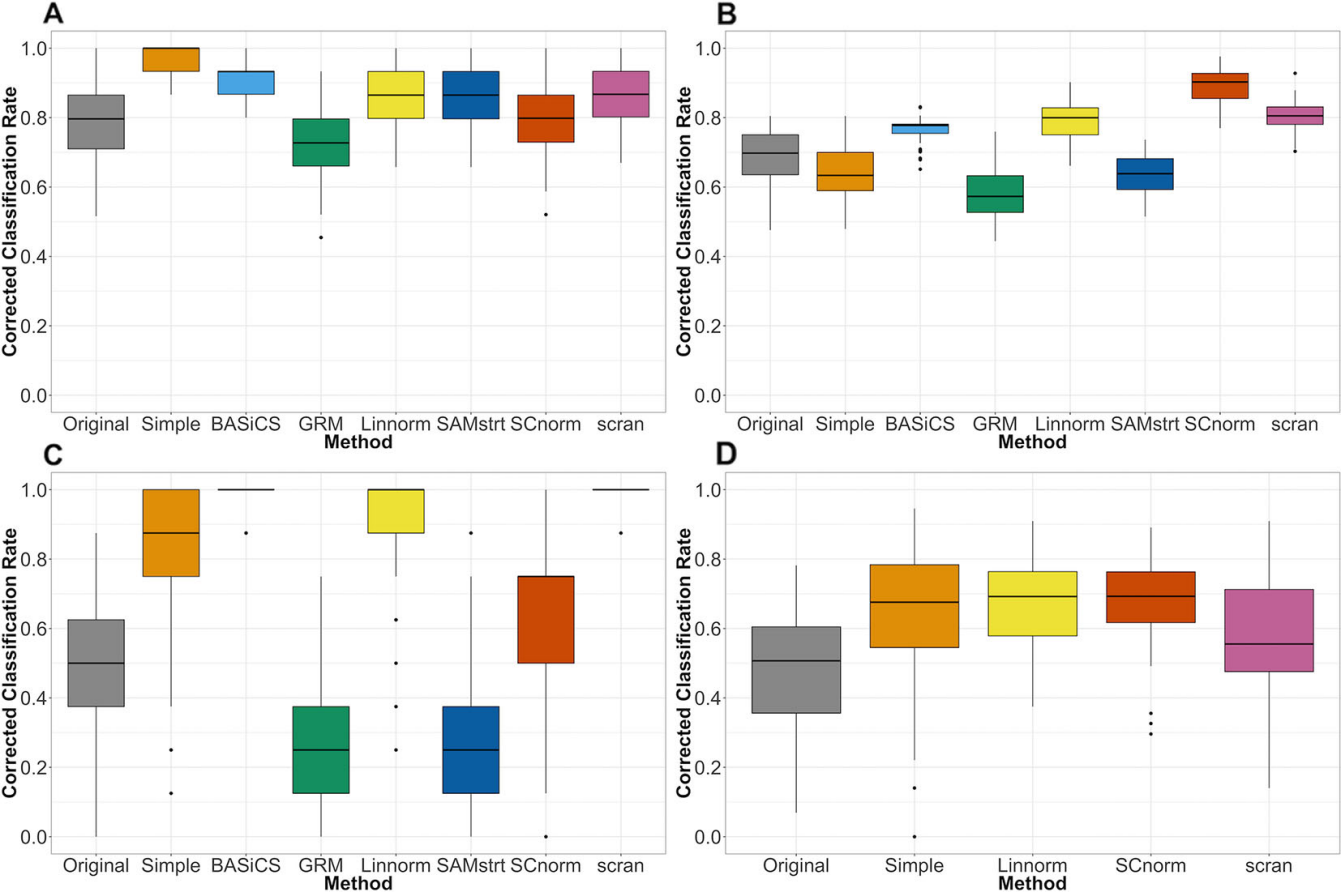
Comparison of normalization methods using the Cohen's kappa statistic. Statistics measuredover 100 random samples. **(A)** Mouse embryonic data **(B)** Mouse lung data **(C)** 2-fold change simulated data **(D)** Human embryonic data.

The classification results for the mouse embryonic data set (**Figure 6**) suggest that the classification rate is improved for the normalized data compared to the rate for the raw data set (i.e., before normalization), with BASiCS slightly outperforming other methods. Classification results for the mouse lung data set (**Figure 6**) show that SCnorm surpasses other methods as the most effective, while both GRM and SAMstrt's results are inferior to those methods for the raw data. For the human embryonic data (**Figure 6**), SCnorm is again the top performer, though Linnorm is close behind. However, it is notable that the simple normalization approach appeared to outperform other methods for the mouse embryonic data set, though it was significantly less effective for the mouse lung data set.

For the simulation study, the differences in performance among normalization methods grow more pronounced, with certain methods performing perfect or near-perfect classification while others fail to improve performance over the raw data (**Figure 6**). The BASiCS and scran methods show the strongest performance for the 2-fold change data set, followed by Linnorm and SCnorm. In comparison, both GRM and SAMstrt are not as effective as other methods when subjected to the simulated data. Consistent conclusions can be obtained for the 5-fold change data set (**Supplementary Figure 15**), where BASiCS and scran remain the top methods with the addition of Linnorm as a close competitor.

**Figure 7:**
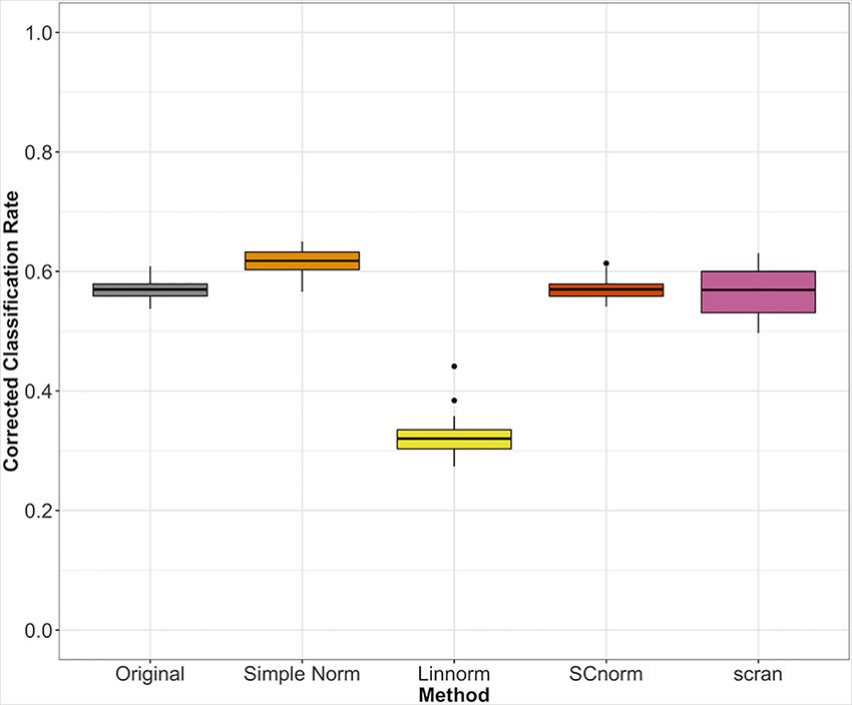
Comparison of normalization methods using the Cohen's kappa statistic for the PBMC data set.

For datasets without spike-ins, the three methods included (Linnorm, SCnorm, scran) had comparable performance, with the exception of Linnorm underperforming for the PBMC data set (**Figure 7**). However, particularly for the PBMC data set, there does not appear to be a noticeable improvement by any of the methods over the original data set.

### Differential Expression Analysis

**Figure 8:**
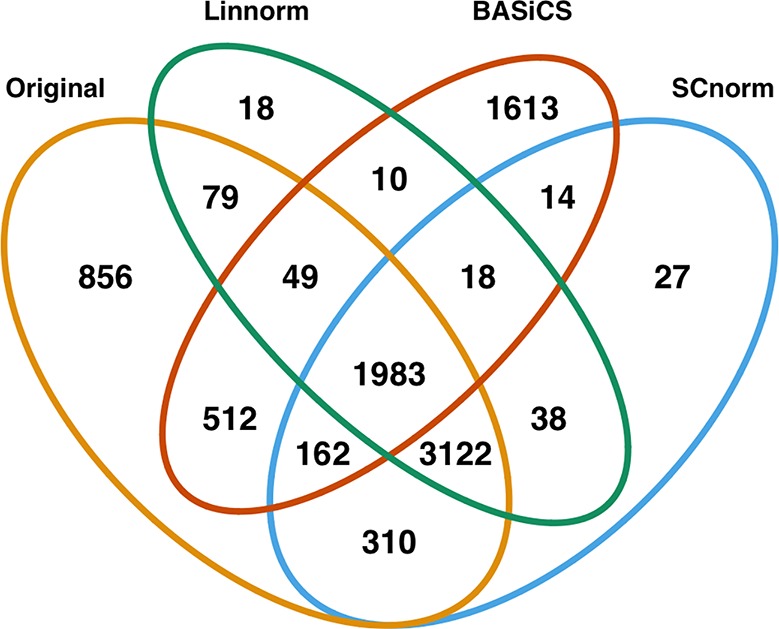
Venn diagram of all DE genes detected at 0.05 significance level for the mouse embryonic dataset.

**Figure 9:**
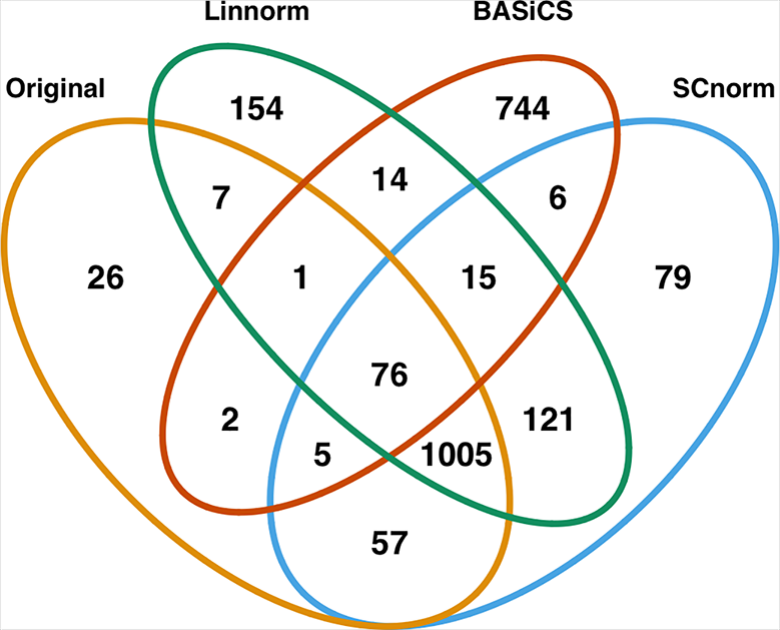
Venn diagram of all DE genes detected at 0.05 significance level for the mouse lung data set.

**Figure 10:**
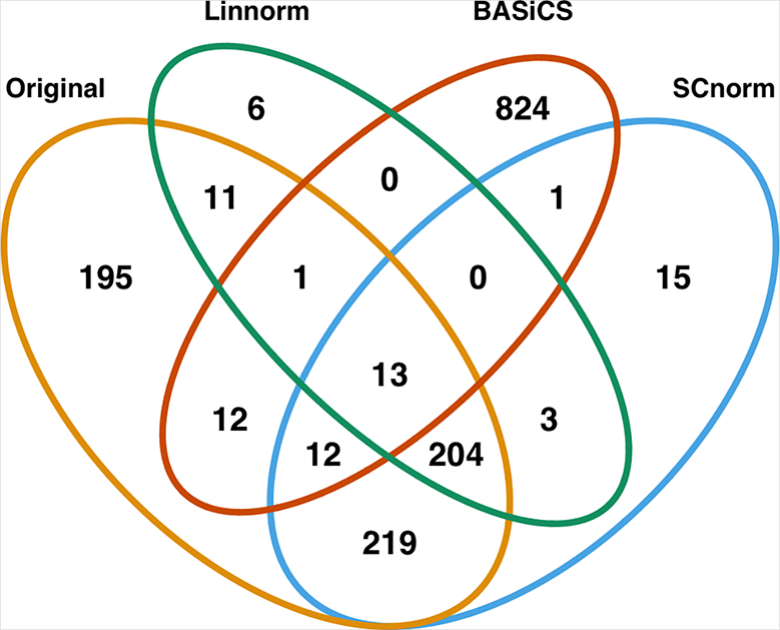
Venn diagram of all DE genes detected at 0.05 significance level for the human embryonic data set.

In addition to visualization and classification, we consider the ability of each normalization method to identify highly differentially expressed (DE) genes. To assess the performance of DE analysis, we consider p-values adjusted for multiple testing by the Benjamini–Hochberg (BH) procedure (Benjamini and Hochberg, 1995) at a 0.05 significance level, generated through the use of R's DESeq2 package. The following Venn diagrams (**Figures 8**–**10**) display the number of DE genes detected at this level of significance for several normalization methods applied to each data set. As seen in **Table 4**, none of the three methods featured is consistently the best at identifying DE genes across all data sets.

**Table 4:** Number of DE genes detected by DESeq2 at the 0.05 significance level for the original data and data normalized by three methods (Linnorm, BASiCS, and SCnorm) for three data sets.

	Original	Linnorm	BASiCS	SCnorm
Mouse Embryonic	7073	5317	4361	5674
Mouse Lung	1179	1393	863	1364
Human Embryonic	667	856	863	467

## Discussion and Conclusion

Based on dimension reduction followed by visualization and KNN classification for the seven normalization methods, we can evaluate/access their effectiveness, as seen in **Table 5**. BASiCS showed noticeable improvement over the raw data across spike-in real data sets and simulated sets, performing best among all methods on the mouse embryonic and simulated data sets. However, it was not very effective on the mouse lung data set, and had a longer execution time than many other methods. SCnorm was the most effective method for both the mouse lung data set and the human embryonic data set, in addition to being one of the best methods in terms of visualization. However, it did not perform well for the mouse embryonic data set, and its execution time was also the longest of all methods compared. Due to its implementation of clustering analysis to determine the optimal number of gene groups, execution time could vary considerably for other data sets. Linnorm and scran were extremely quick to execute for their quality of normalization, though neither was exclusively the top method for any data set. Both methods demonstrated consistent performance across all data sets, with scran matching BASiCS for simulated data and Linnorm surpassing scran for the human data set. While SAMstrt was nearly as fast a method as scran, it did not show improvement for any of the data sets except the mouse embryonic data, and even then, other methods outperformed it. GRM resulted in some unusual outliers and frequent misclassifications for the spike-in real data sets, and it consistently lagged behind other methods for the simulated data sets.

**Table 5:** Comparison of normalization methods used, including execution time (rounded to nearest five seconds), visualization, and classification rate across all data sets.

Category	BASiCS	GRM	Linnorm	SAMstrt	SCnorm	scran	Simple Norm
Mouse Embryonic Data (sec)	230	35	<5	<5	760	<5	<5
Mouse Lung Data (sec)	510	60	<5	5	1180	<5	<5
Mouse Embryonic Sim Data (sec)	110	15	<5	<5	110	<5	<5
Human Embryonic Data (sec)	_	_	<5	_	370	<5	<5
Classification (Spike-In Genes)	***	*	**	*	***	**	**
Visualization (Spike-In Genes)	**	*	**	*	**	**	**
Classification (Non-Spike-In)	_	_	*	_	**	**	**
Visualization (Non-Spike-In)	_	_	*	_	**	*	**

A ‘***' indicates that the method showed exemplary performance overall, a ‘**' indicates satisfactory performance overall, and a ‘*' indicates some shortcoming in performance compared to other methods. ‘_' indicates that the method is not applicable for this type of data. Two variants exist for the Mouse Embryonic Sim Data, but they had negligible differences in execution time.

In the GRM method, an individual model is constructed for each cell at a time. Thus, the method does not incorporate the relationship across cells under the same condition, potentially losing valuable information. SAMstrt appears to be strongly dependent on the quantity of spike-ins, which may explain why its performance for classification is not consistent across the data sets tested. BASiCS's ability to incorporate more than just spike-in genes for its calculations allows it to obtain more consistent results than other spike-in-based approaches, though the process takes comparatively longer to execute. SCnorm's versatile nature allows it to automatically adjust the number of necessary groups to the data, pursuing more finely tuned results at the expense of extra execution time. It also includes an additional automatic filtering step that allows it to focus on the genes most relevant to each condition, resulting in better classification. Though several methods are quick to execute, Linnorm is potentially the fastest among these due to its implementation with time complexity *O*(n*log(*n*)), allowing easy scaling to larger datasets compared to other methods and accounting for its swift performance. Both Linnorm and scran are written in C++ and implemented in R, contributing to their swift execution. As may be expected, no single method will be the clear choice for every data set, but the strengths and weaknesses of each are useful knowledge when deciding which approach to employ. The exceptionally low runtime of scran or Linnorm may be enough to warrant its use if the size of the data is a concern, while SCnorm often provides better performance if execution time is not a critical factor or if spike-ins are not available in the study. BASiCS is also a sound option when spike-in genes are available.

Despite these slight advantages, comparisons to the simple normalization process built in to Seurat reveals that even exceptional methods do not greatly distinguish themselves from a more straightforward normalization approach. The limitations of these normalization methods on the 10X data set depicted also highlights the need for a method that can appropriately adjust to this new format and its properties. Additionally, this study focuses primarily on visualization and classification methods, with a brief discussion of differential expression. Performance for these methods may differ when applied to additional types of analysis, such as trajectory inference.

## Data Availability Statement

Publicly available datasets were analyzed in this study. This data can be found here: GSE29087, GSE52583, GSE64016 from the NCBI GEO database, and from 10x Genomics (http://support.10xgenomics.com/single-cell/datasets).

## Author Contributions

NL, DR, and LA contributed to the overall design of this study. NL performed the statistical analysis and wrote the initial draft of this manuscript. DR and LA contributed additions and revisions to the statistical analysis. All authors revised, proofread, and approved the submitted manuscript.

## Funding

This work was supported by the National Institutes of Health (GM084905) and United States Department of Agriculture (ARZT-1360830-H22-138).

## Conflict of Interest

The authors declare that the research was conducted in the absence of any commercial or financial relationships that could be construed as a potential conflict of interest.
