# Supplementary material for: Normalization Methods on Single-Cell RNA-seq Data: An Empirical Survey
Source: Front Genet. 2020 Feb 7;11:41. doi: 10.3389/fgene.2020.00041 (PMC7019105; doi:10.3389/fgene.2020.00041)
Supplement: Supplementary file 1 [file DataSheet_1.docx]

Supplementary Material

# Basic Cleaning

Before applying each normalization method, all data sets were first subjected to a basic cleaning process. During cleaning, all cells with minimum library size below 1000 counts were removed, as were any genes not present in at least five cells. After the cleaning the mouse embryonic data contained 46 ES cells and 44 MEF cells, and the mouse lung data contained 45 E14.5 cells, 26 E16.5 cells, 80 E18.5 cells, and 35 adult cells. Additionally, the PBMC data included 2649 of the original 2700 cells, including the seven largest cell groups identified. The simulation data and human embryonic data incurred no changes after cleaning. All of the data sets were normalized at the original scale but visualized at the log-transformed scale to better represent the large range of possible counts for genes. We used log(count+1) transformation to prevent undefined values for zero counts.

# Visualization

All t-SNE plots for real data sets were run with perplexity of 25, theta of 0.5, and 2000 iterations maximum. As perplexity cannot exceed group size, the simulated data sets required lower perplexity; in these cases, t-SNE was run with perplexity of 15 instead.

## Supplementary Figures


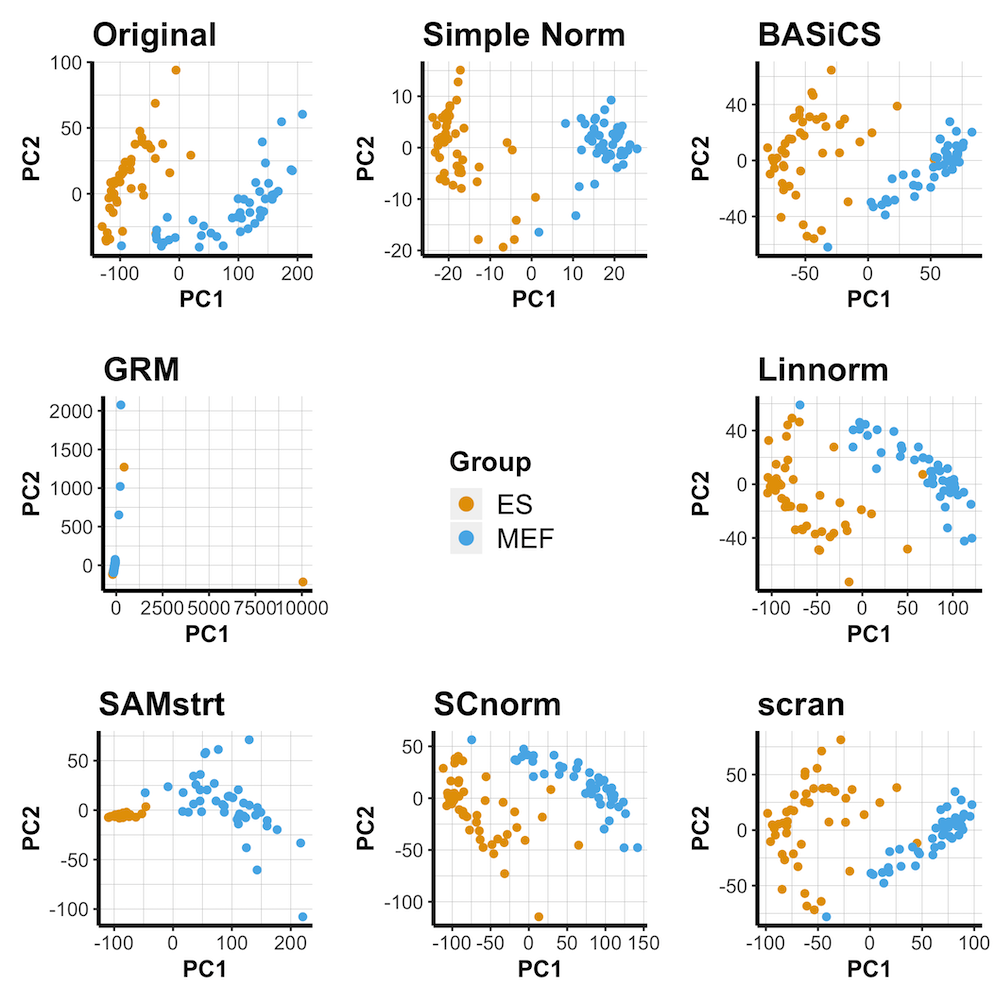


**Supplementary Figure 1.** PCA plots of the mouse embryonic data set under various normalization methods.


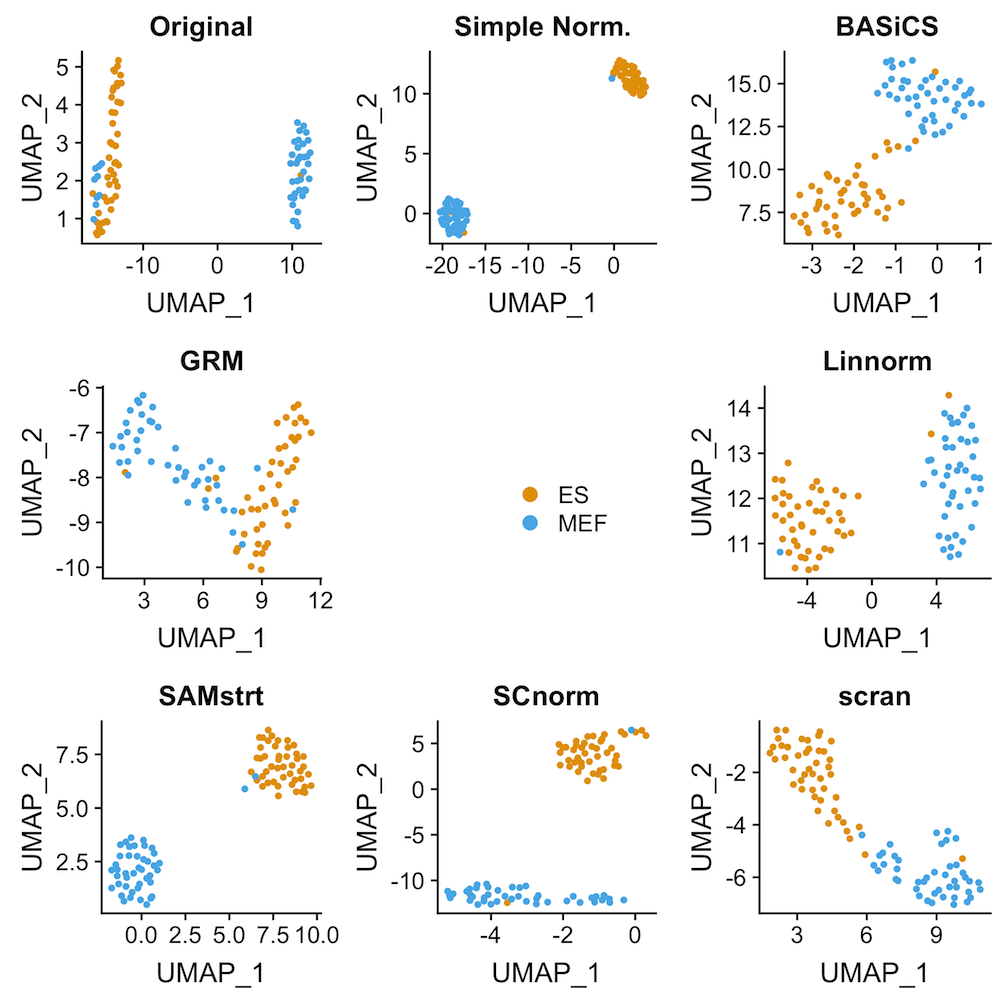


**Supplementary Figure 2**: UMAP plots of the mouse embryonic data set under various normalization methods.

**
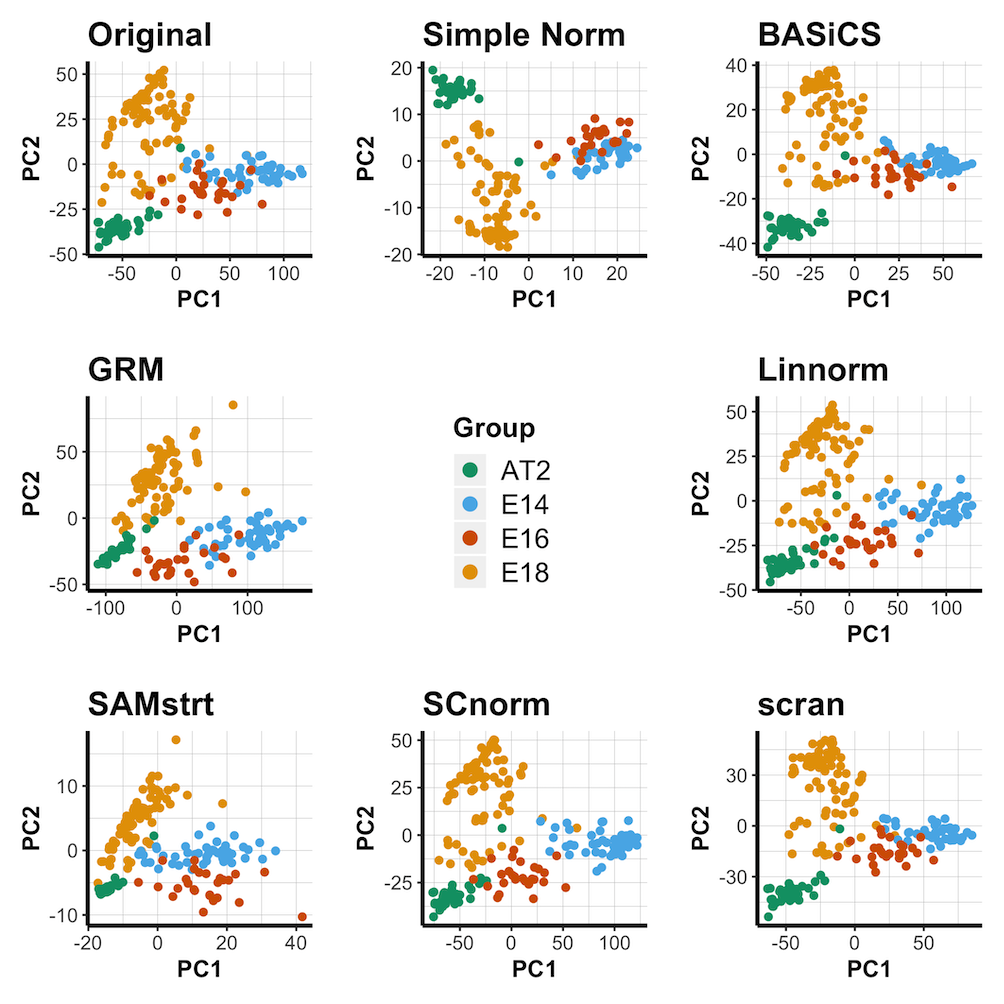
**

**Supplementary Figure 3**: PCA plots of the mouse lung data set under various normalization methods.


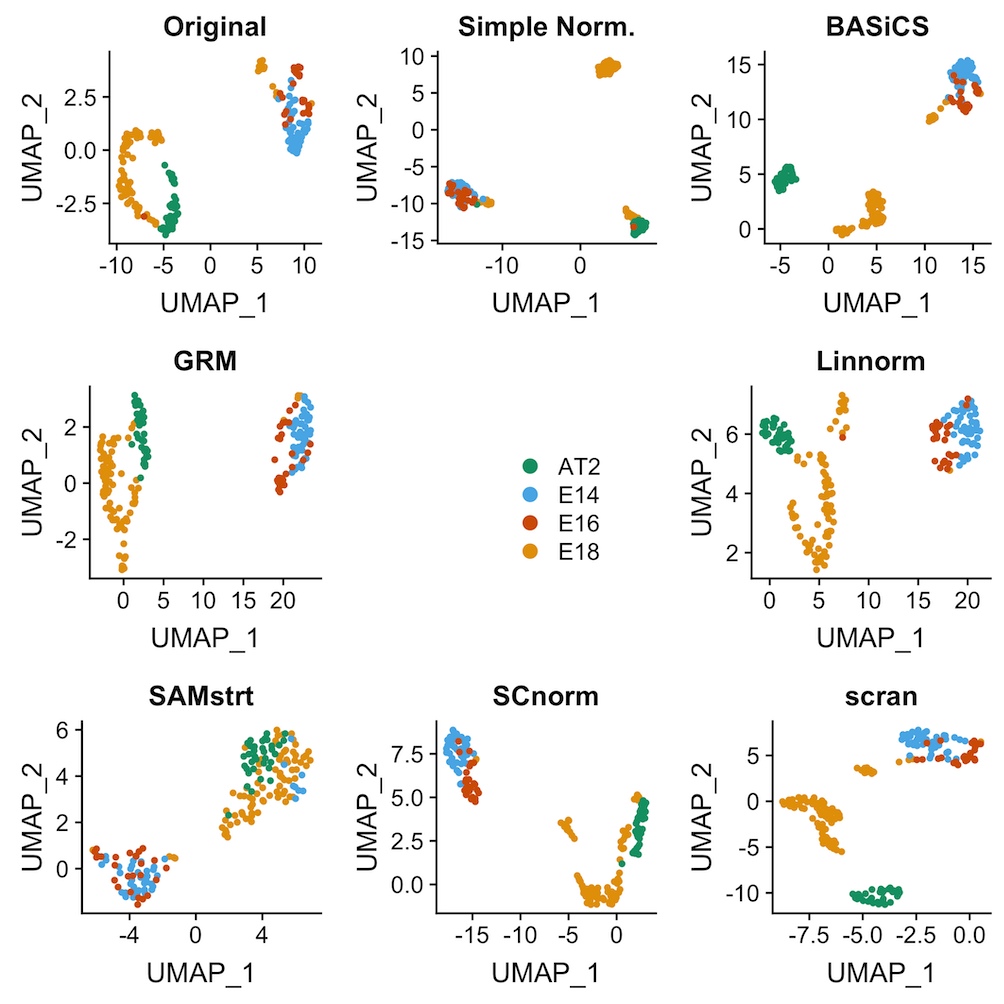


**Supplementary Figure 4**: UMAP plots of the mouse lung data set under various normalization methods.


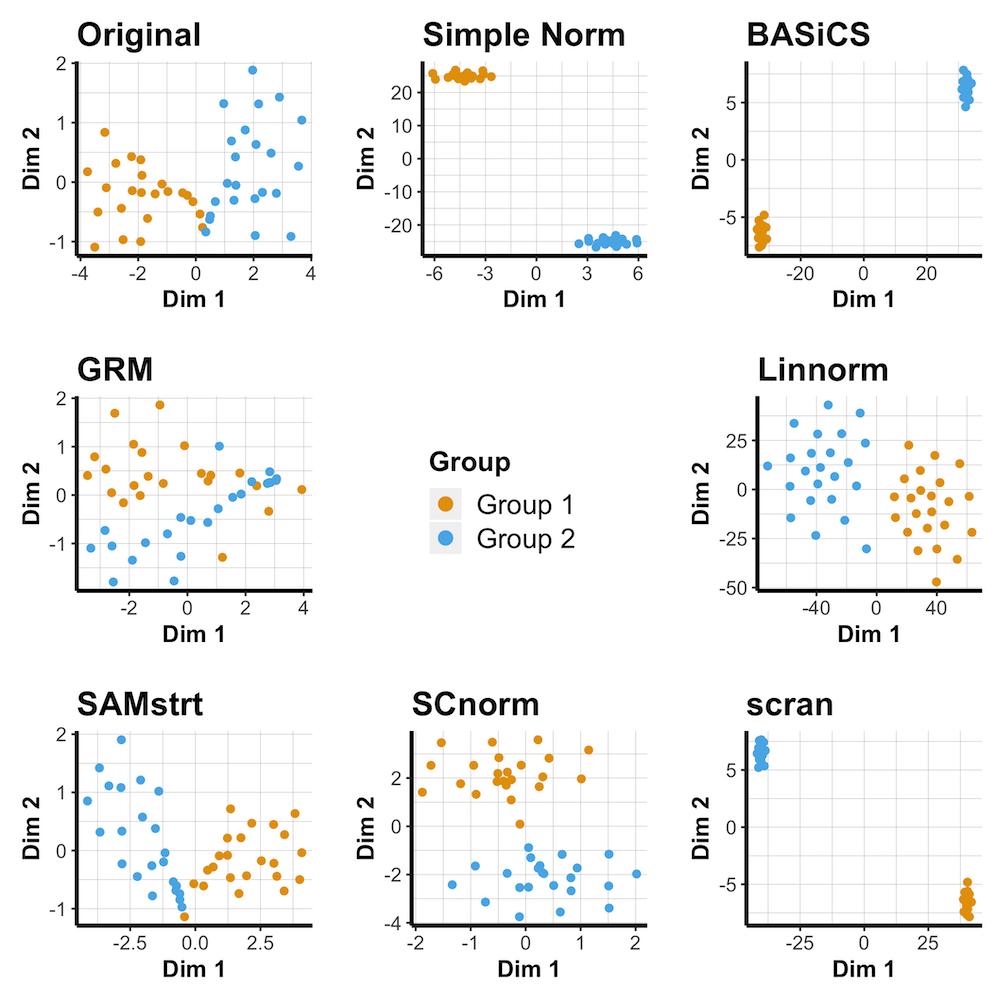


**Supplementary Figure 5:** t-SNE plots for the simulated data set with 5-fold change difference under various normalization methods.

**
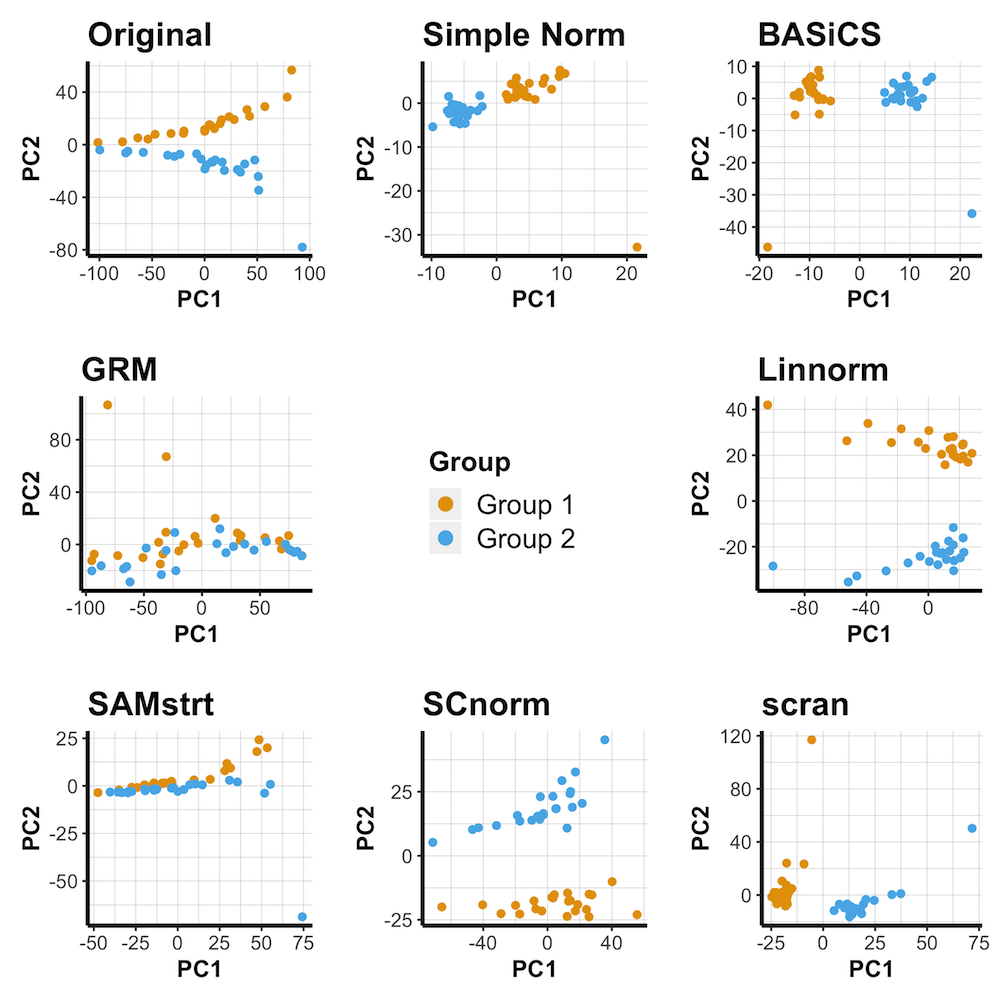
**

**Supplementary Figure 6**: PCA plots of the simulated data set with 2-fold change difference under various normalization methods.


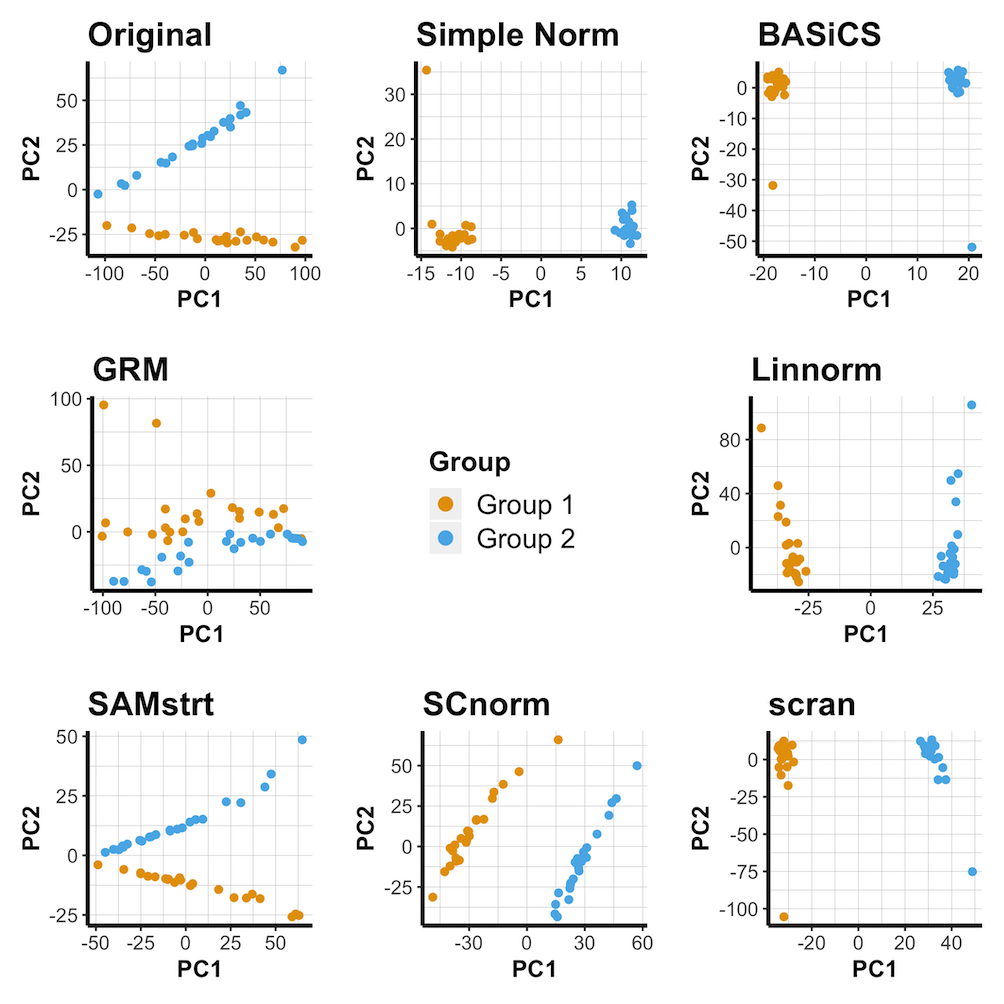


**Supplementary Figure 7**: PCA plots of the simulated data set with 5-fold change difference under various normalization methods.


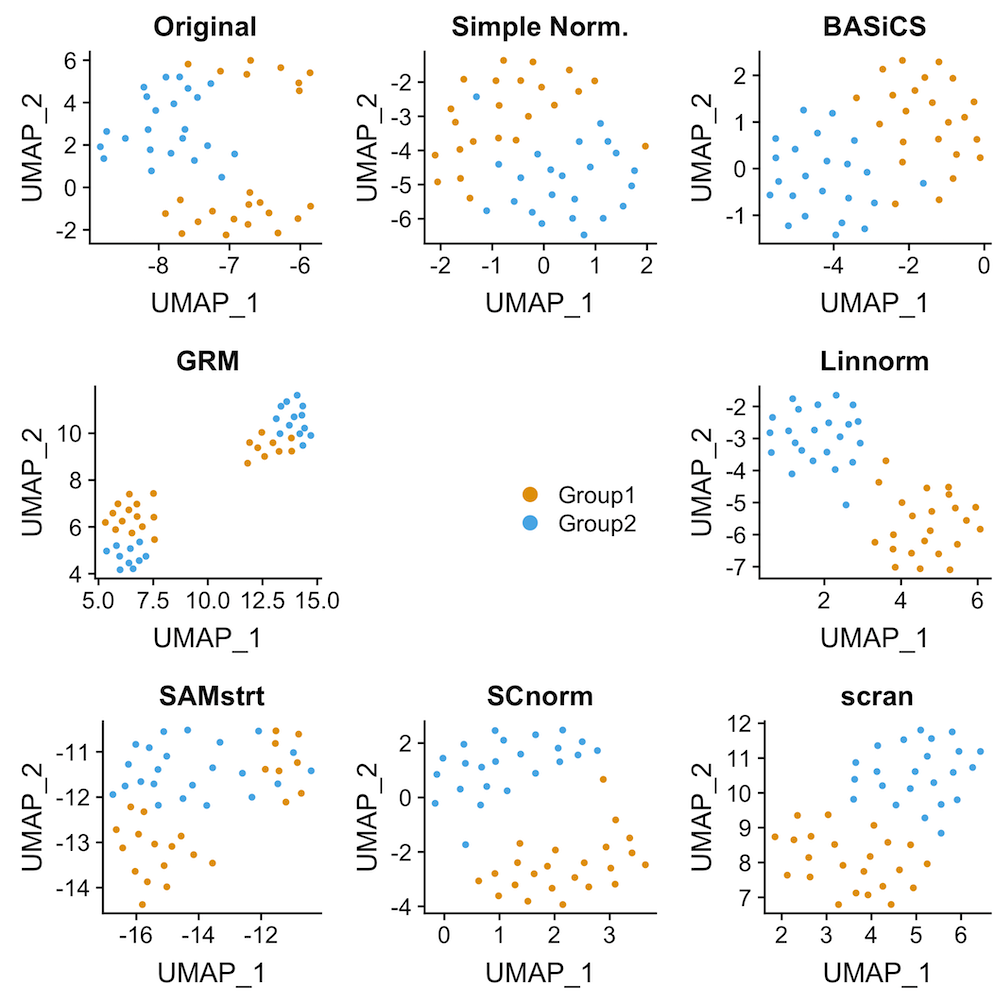


**Supplementary Figure 8**: UMAP plots of the simulated data set with 2-fold change difference under various normalization methods.


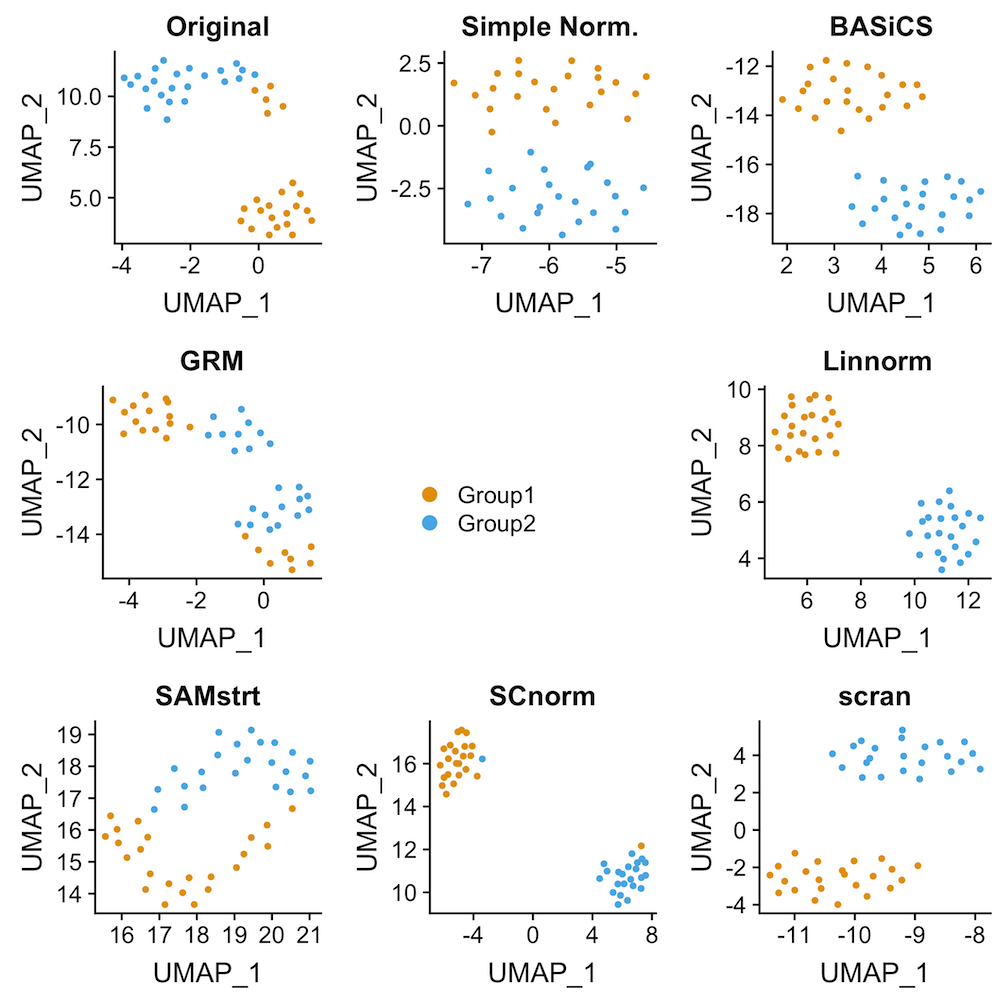


**Supplementary Figure 9**: UMAP plots of the simulated data set with 5-fold change difference under various normalization methods.


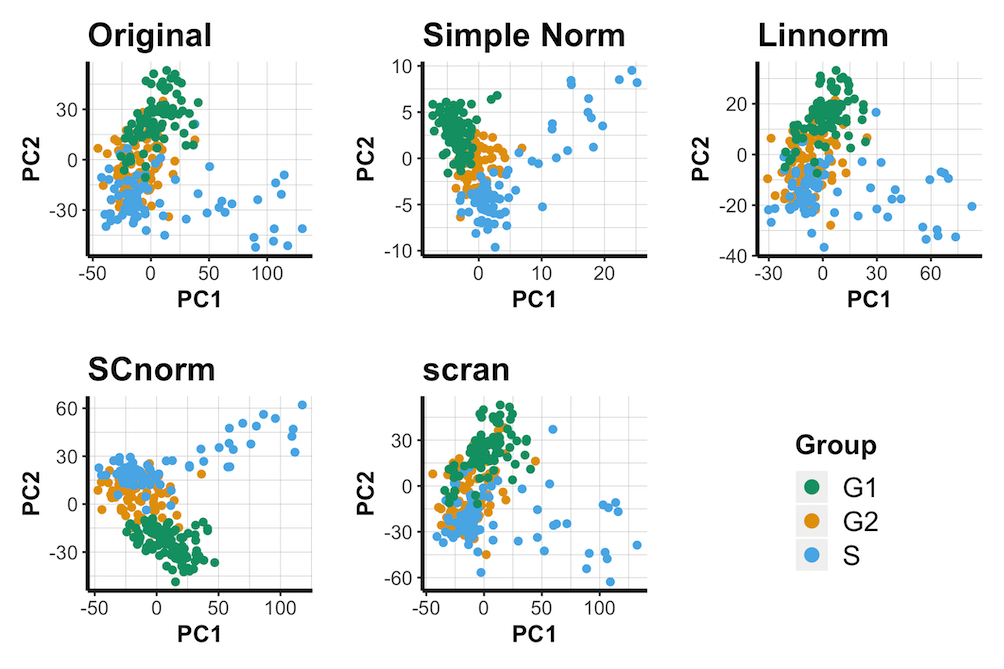


**Supplementary Figure 10**: PCA plots of the human embryonic data set under various normalization methods.


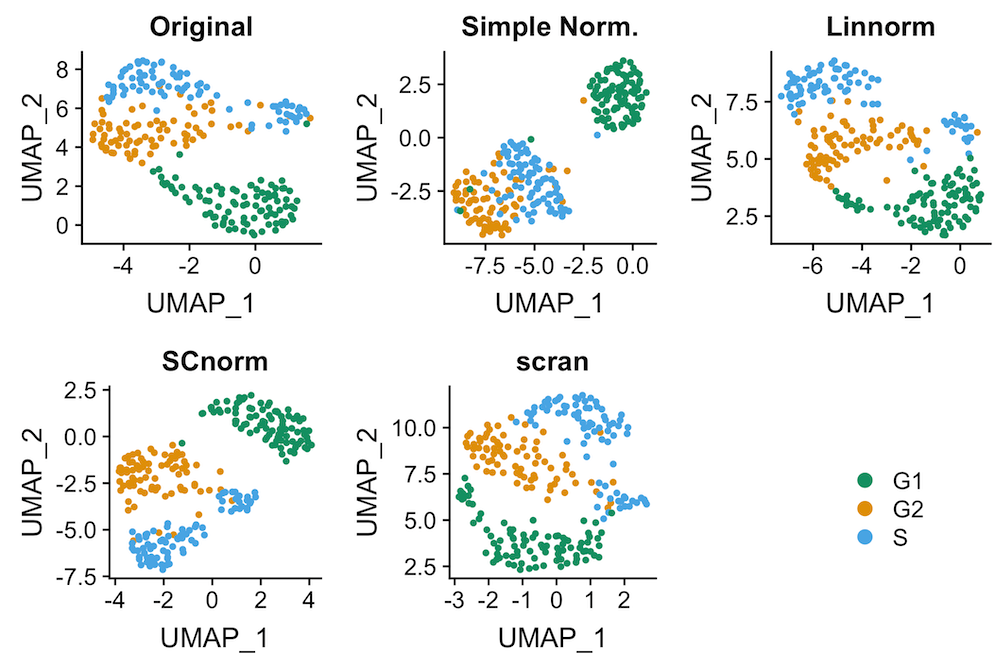


**Supplementary Figure 11**: UMAP plots of the human embryonic data set under various normalization methods.

**
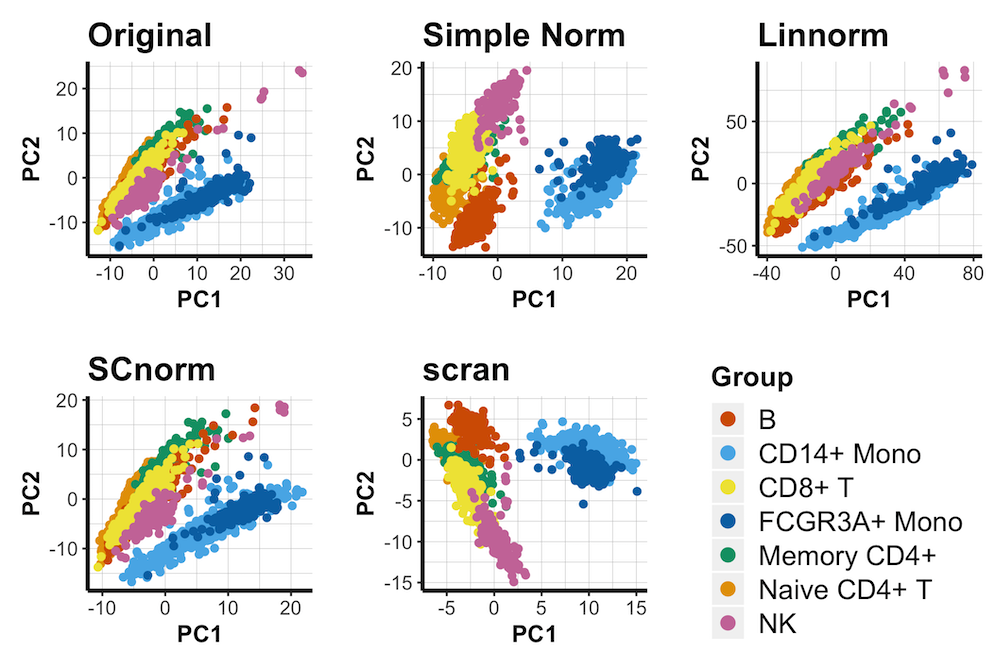
**

**Supplementary Figure 12**: PCA plots of the 10X PBMC data set under various normalization methods.


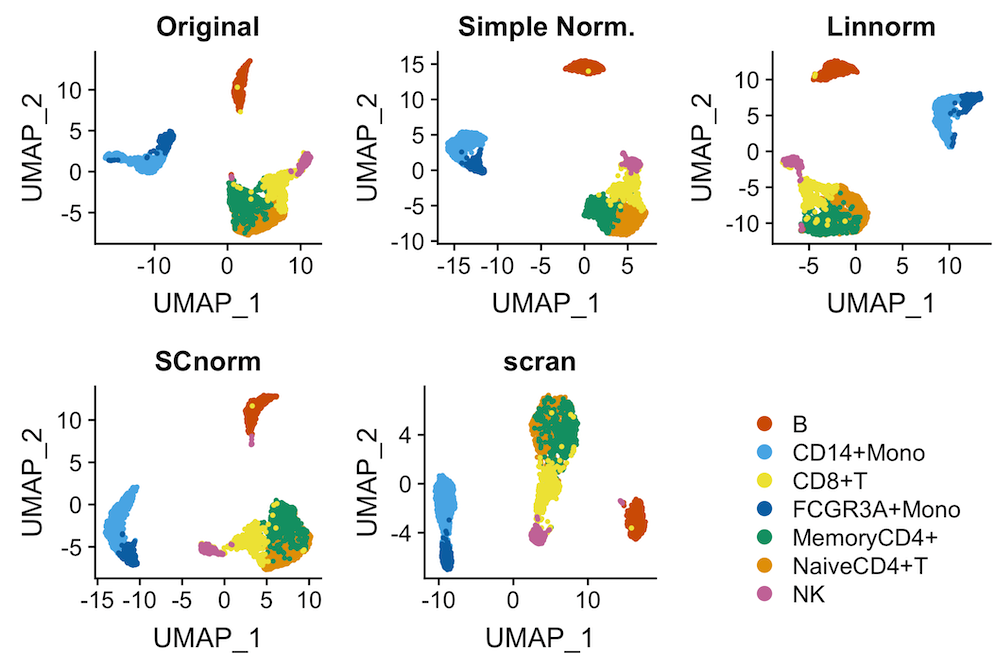


**Supplementary Figure 13**: UMAP plots of the 10X PBMC data set under various normalization methods.


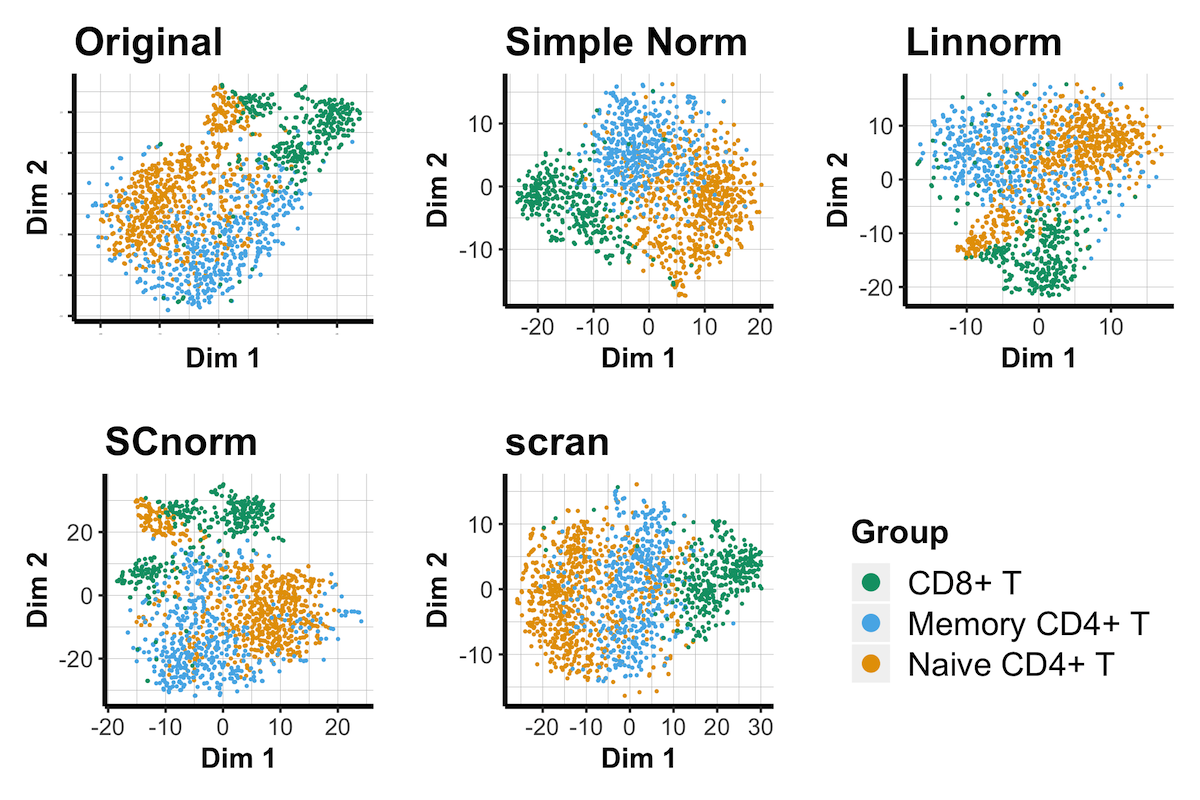


**Supplementary Figure 14**: t-SNE plot for a subset of the PBMC data consisting of three different T-cell types.

# Classification Analysis

After randomly selecting and combining one-third of the cells from each condition to serve as a training set, we then performed KNN on the remaining cells to generate a confusion matrix for predicted group versus true group. Cohen’s kappa statistic, a corrected classification rate, is then calculated for comparing the performance of normalization methods. We repeated this procedure 100 times for each dataset.

## Supplementary Plots


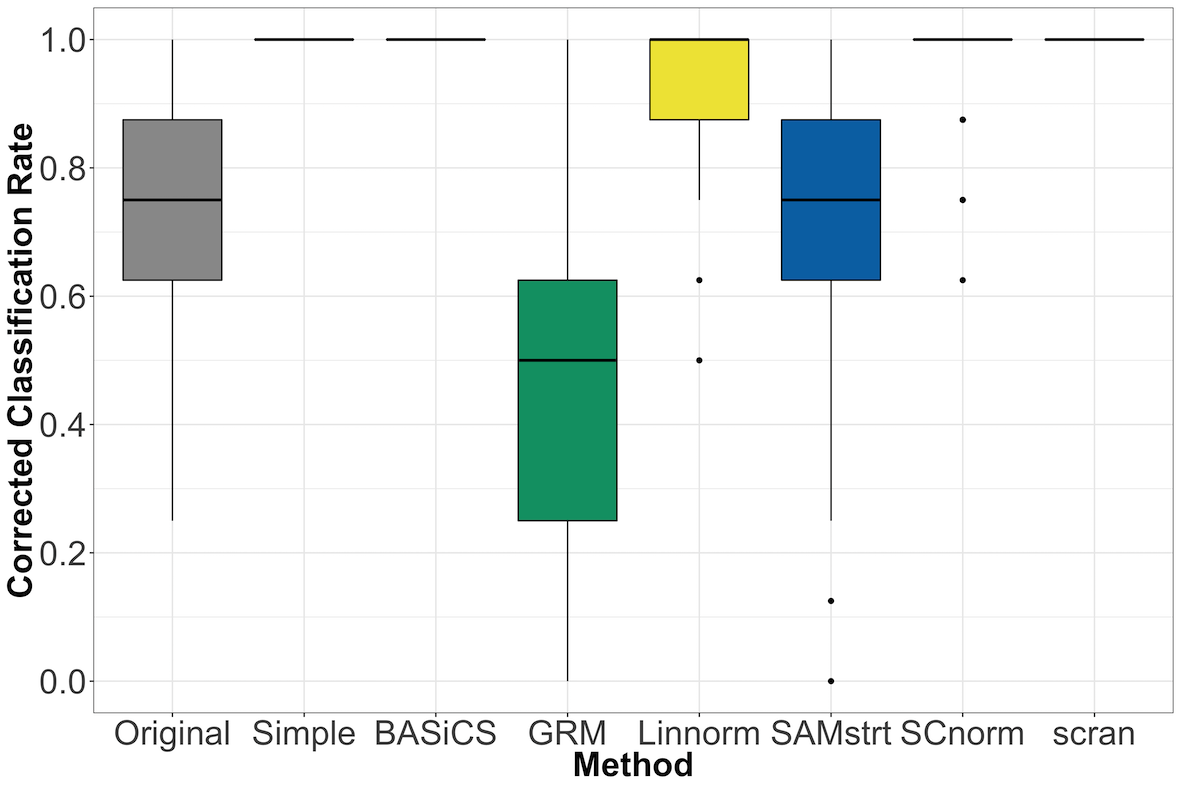


**Supplementary Figure 15**: Comparison of corrected classification rate (Cohen’s kappa statistic) under various normalization methods on the 5-fold change simulated data set. Cohen’s kappa statistic measured over 100 random samples.

# Bulk Sequencing Comparison

Visualization and classification analysis for the two bulk-based methods included here suggests that neither method is able to reach the performance seen by the majority of single-cell-based methods.


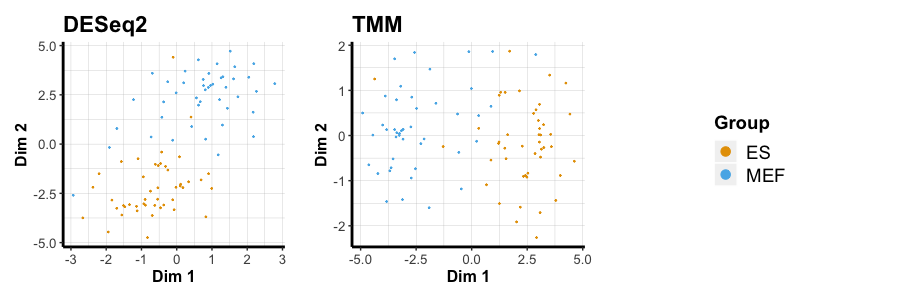


**Supplementary Figure 16:** t-SNE plots for the mouse embryonic data set under bulk-based normalization methods.


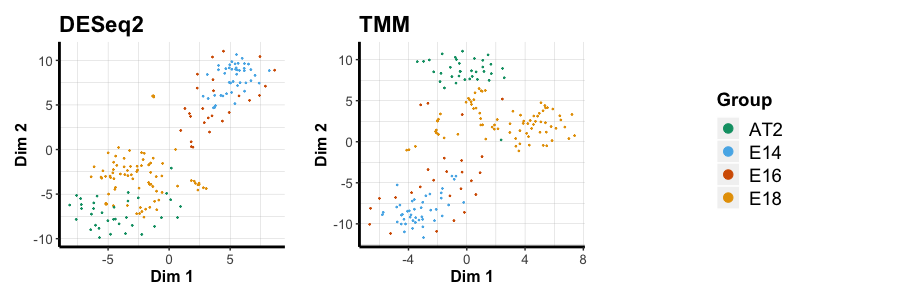


**Supplementary Figure 17:** t-SNE plots for the mouse lung data set under bulk-based normalization methods.


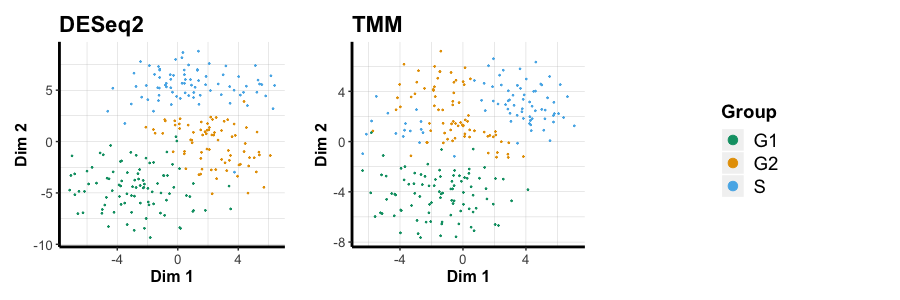


**Supplementary Figure 18:** t-SNE plots for the human embryonic data set under bulk-based normalization methods.


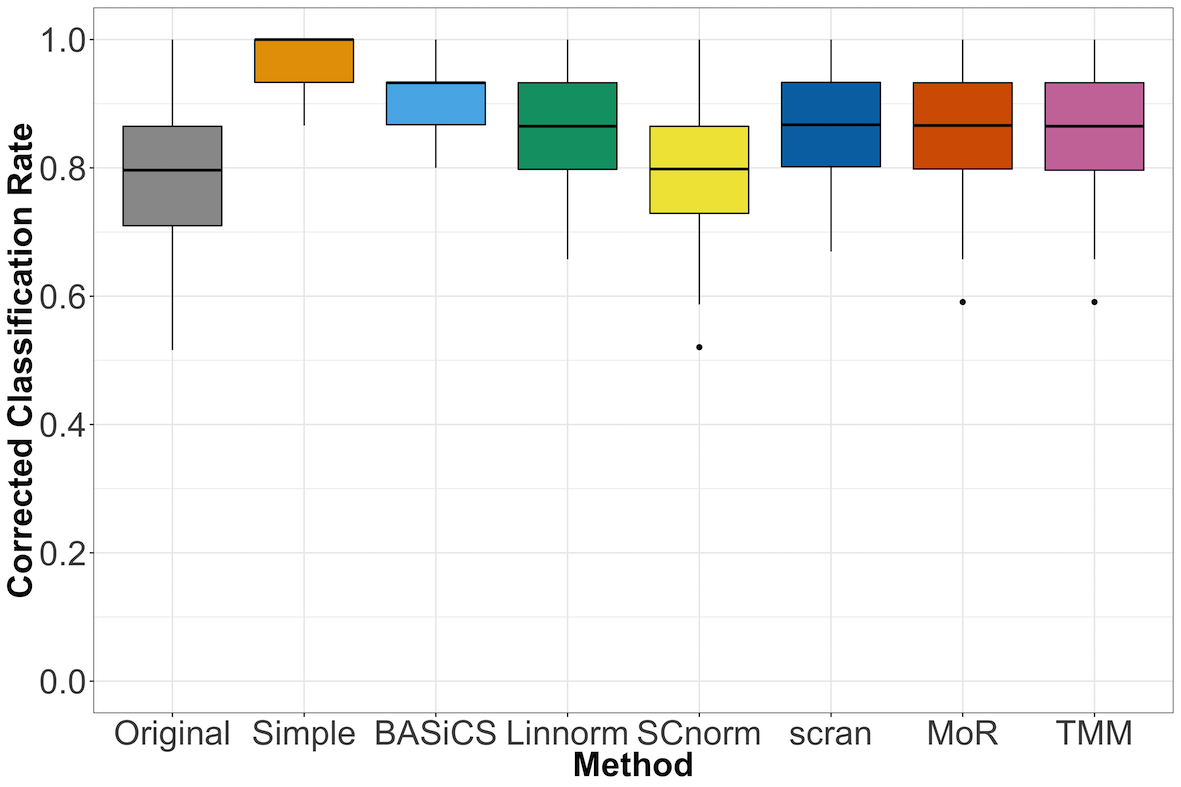


**Supplementary Figure 19:** Comparison of corrected classification rate (Cohen’s kappa statistic) under various normalization methods on the mouse embryonic data set, including bulk-based normalization methods.


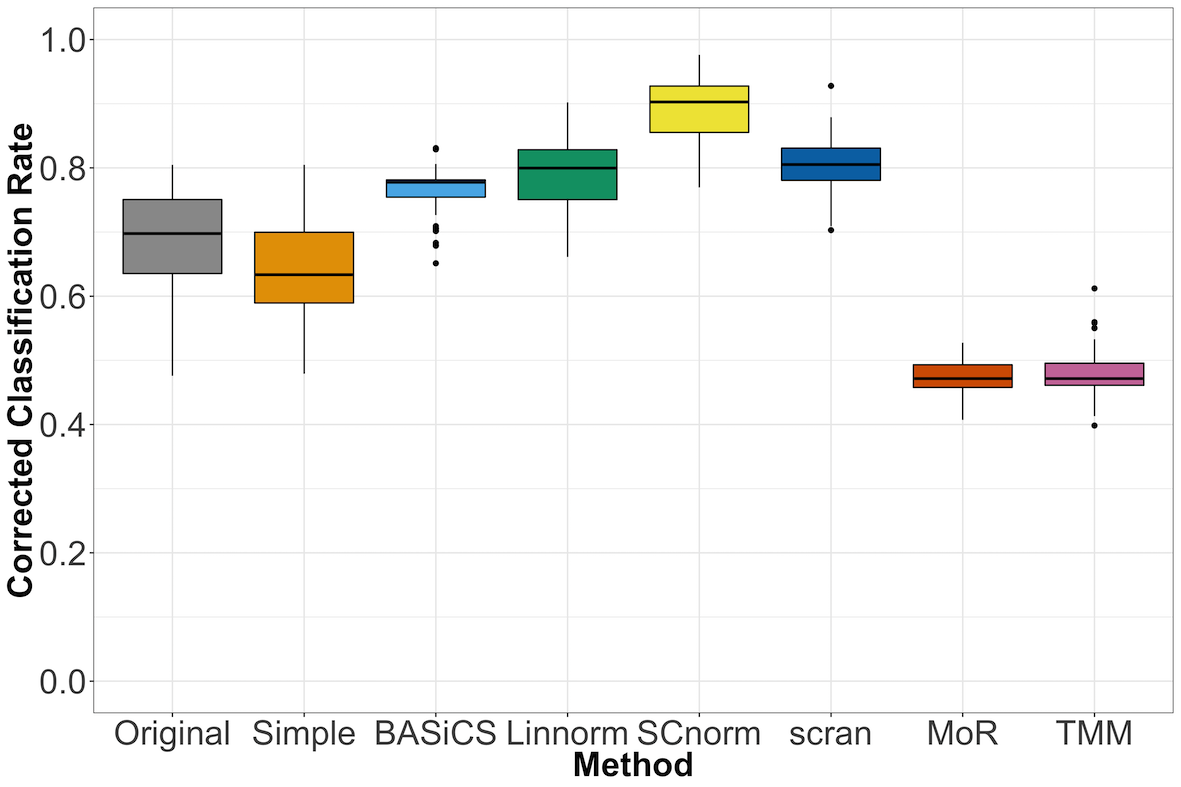


**Supplementary Figure 20:** Comparison of corrected classification rate (Cohen’s kappa statistic) under various normalization methods on the mouse lung data set, including bulk-based normalization methods.


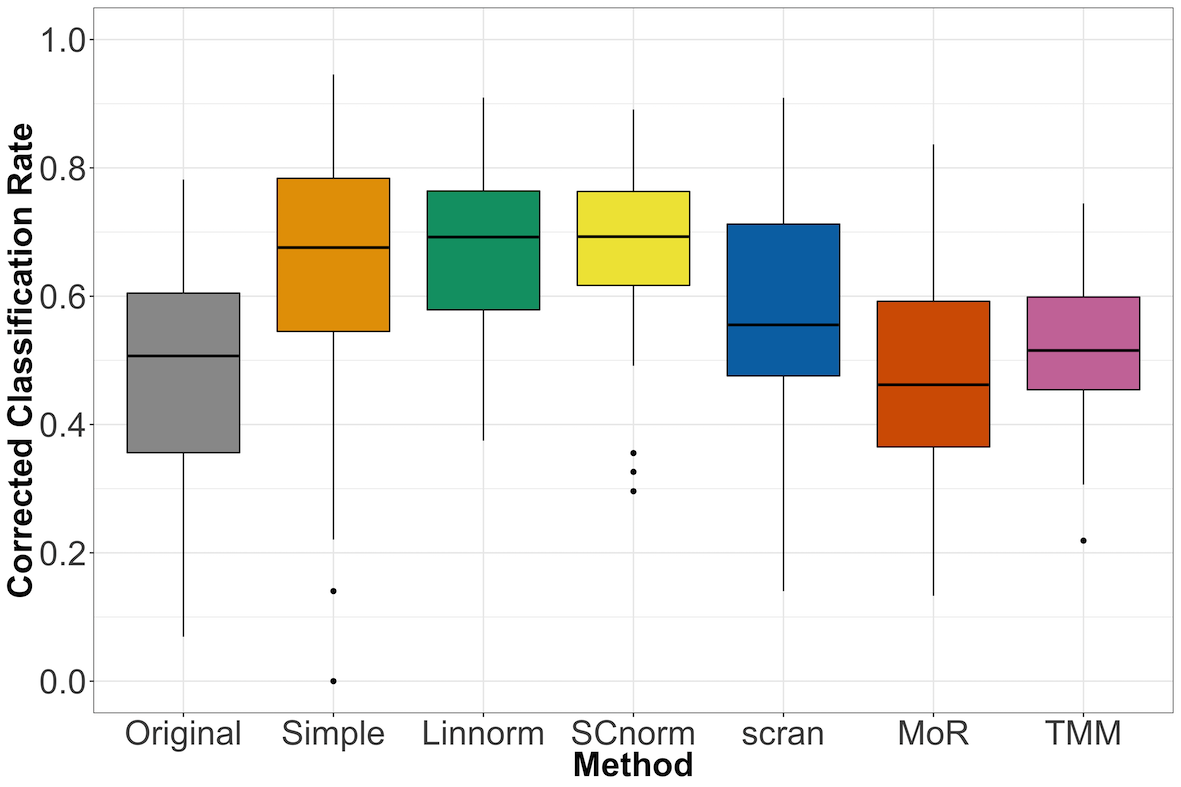


**Supplementary Figure 21:** Comparison of corrected classification rate (Cohen’s kappa statistic) under various normalization methods on the human embryonic data set, including bulk-based normalization methods.
