# Supplementary material for: Normalization Methods on Single-Cell RNA-seq Data: An Empirical Survey
Source: Front Genet. 2020 Feb 7;11:41. doi: 10.3389/fgene.2020.00041 (PMC7019105; doi:10.3389/fgene.2020.00041)
Supplement: Supplementary file 2 [file Table_1.docx]

**Table 1.** Summary of data sets used in this review.

| **Data Type** | **Author** | **Year** | **Protocol** | **Platform** | **# genes after cleaning** | **# cells after cleaning** |
| --- | --- | --- | --- | --- | --- | --- |
| Mouse embryonic stem cells | Islam et al. | 2011 | AbGene Thermo-Fast 96 | Illumina Genome Analyzer IIx | 11430 | 90 |
| Mouse lung cells | Treutlein et al. | 2014 | Fluidigm C1 | Illumina HiSeq 2000 | 12073 | 186 |
| Human embryonic data | Leng et al. | 2015 | Fluidigm C1 | Illumina HiSeq 2500 | 19084 | 247 |
| PBMC data | Zheng et al. | 2017 | 10X Genomics | GemCode | 13714 | 2649 |
