# Supplementary material for: Normalization Methods on Single-Cell RNA-seq Data: An Empirical Survey
Source: Front Genet. 2020 Feb 7;11:41. doi: 10.3389/fgene.2020.00041 (PMC7019105; doi:10.3389/fgene.2020.00041)
Supplement: Supplementary file 3 [file Table_2.docx]

**Table 2**. Summary of normalization methods, including the basic description and whether the method uses spike-in genes information.

| **Method** | **Author** | **Year** | **Spike-ins** | **Model Description** |
| --- | --- | --- | --- | --- |
| SAMstrt | Katayama et al | 2013 | Yes | Poisson resampling and non-parametric statistics |
| BASiCS | Vallejos et al | 2015 | Yes | Use spike-ins for hierarchical Poisson/Gamma model for technical variability. Expand model to incorporate biological genes with new Poisson model |
| GRM | Ding et al | 2015 | Yes | Gamma regression model from spike-ins |
| Simple Norm. | Satija et al | 2015 | No | Divide gene counts for cells, then multiply by scale factor and apply a log(x+1) transformation to the result (included in the Seurat package as NormalizeData) |
| scran | Lun et al | 2016 | No | Deconvolution of size factors from constructed linear system. Form pools of cells and normalize using summed expression values |
| SCnorm | Bacher et al | 2017 | Optional | Quantile based model for log sequencing depth. |
| Linnorm | Yip et al | 2017 | Optional | Linear models defined with a normalization strength coefficient to update means. Focuses on stable genes to perform normalization |
