# Supplementary material for: Normalization Methods on Single-Cell RNA-seq Data: An Empirical Survey
Source: Front Genet. 2020 Feb 7;11:41. doi: 10.3389/fgene.2020.00041 (PMC7019105; doi:10.3389/fgene.2020.00041)
Supplement: Supplementary file 4 [file Table_3.docx]

**Table 3**. A list of source packages for downloading.

| **Package** | **Source** |
| --- | --- |
| SAMstrt v.0.99.0 | (https://github.com/shka/R-SAMstrt/archive/0.99.0.tar.gz) |
| BASiCS v.1.0.1 | Bioconductor (http://www.bioconductor.org) |
| GRM v.0.2.1 | (http://wanglab.ucsd.edu/star/GRM/) |
| scran v.1.6.9 | Bioconductor (http://www.bioconductor.org) |
| SCnorm v.1.0.0 | Bioconductor (http://www.bioconductor.org) |
| Linnorm v.2.2.0 | Bioconductor (http://www.bioconductor.org) |
| Seurat v.3.0.0 | CRAN (https://CRAN.R-project.org/package=Seurat) |
