# Supplementary material for: Normalization Methods on Single-Cell RNA-seq Data: An Empirical Survey
Source: Front Genet. 2020 Feb 7;11:41. doi: 10.3389/fgene.2020.00041 (PMC7019105; doi:10.3389/fgene.2020.00041)
Supplement: Supplementary file 5 [file Table_4.docx]

**Table 4.** Comparison of normalization methods used, including execution time (rounded to nearest five seconds), visualization, and classification rate across all data sets. A ‘***’ indicates that the method showed exemplary performance overall, a ‘**’ indicates satisfactory performance overall, and a ‘*’ indicates some shortcoming in performance compared to other methods. ‘_’ indicates that the method is not applicable for this type of data. Two variants exist for the Mouse Embryonic Sim Data, but they had negligible differences in execution time.

| Category | BASiCS | GRM | Linnorm | SAMstrt | SCnorm | scran | Simple Norm |
| --- | --- | --- | --- | --- | --- | --- | --- |
| Mouse Embryonic Data (sec) | 230 | 35 | <5 | <5 | 760 | <5 | <5 |
| Mouse Lung Data (sec) | 510 | 60 | <5 | 5 | 1180 | <5 | <5 |
| Mouse Embryonic Sim Data (sec) | 110 | 15 | <5 | <5 | 110 | <5 | <5 |
| Human Embryonic Data (sec) | _ | _ | <5 | _ | 370 | <5 | <5 |
| Classification (Spike-In Genes) | *** | * | ** | * | *** | ** | ** |
| Visualization (Spike-In Genes) | ** | * | ** | * | ** | ** | ** |
| Classification (Non-Spike-In) | _ | _ | * | _ | ** | ** | ** |
| Visualization (Non-Spike-In) | _ | _ | * | _ | ** | * | ** |
