# Supplementary material for: Normalization Methods on Single-Cell RNA-seq Data: An Empirical Survey
Source: Front Genet. 2020 Feb 7;11:41. doi: 10.3389/fgene.2020.00041 (PMC7019105; doi:10.3389/fgene.2020.00041)
Supplement: Supplementary file 6 [file Table_5.docx]

**Table 5**: Number of DE genes detected by DESeq2 at the 0.05 significance level for the original data and data normalized by three methods (Linnorm, BASiCS, and SCnorm) for three data sets.

|  | **Original** | **Linnorm** | **BASiCS** | **SCnorm** |
| --- | --- | --- | --- | --- |
| Mouse Embryonic | 7073 | 5317 | 4361 | 5674 |
| Mouse Lung | 1179 | 1393 | 863 | 1364 |
| Human Embryonic | 667 | 856 | 863 | 467 |
